# Identification and Characteristics of Fusion Peptides Derived From Enveloped Viruses

**DOI:** 10.3389/fchem.2021.689006

**Published:** 2021-08-23

**Authors:** Camille Lozada, Thomas M. A. Barlow, Simon Gonzalez, Nadège Lubin-Germain, Steven Ballet

**Affiliations:** ^1^ BioCIS, CNRS, CY Cergy-Paris Université, Cergy-Pontoise, France; ^2^ Research Group of Organic Chemistry, Vrije Universiteit Brussel, Brussels, Belgium

**Keywords:** fusion, peptides, enveloped viruses, secondary structures, membranotropic

## Abstract

Membrane fusion events allow enveloped viruses to enter and infect cells. The study of these processes has led to the identification of a number of proteins that mediate this process. These proteins are classified according to their structure, which vary according to the viral genealogy. To date, three classes of fusion proteins have been defined, but current evidence points to the existence of additional classes. Despite their structural differences, viral fusion processes follow a common mechanism through which they exert their actions. Additional studies of the viral fusion proteins have demonstrated the key role of specific proteinogenic subsequences within these proteins, termed fusion peptides. Such peptides are able to interact and insert into membranes for which they hold interest from a pharmacological or therapeutic viewpoint. Here, the different characteristics of fusion peptides derived from viral fusion proteins are described. These criteria are useful to identify new fusion peptides. Moreover, this review describes the requirements of synthetic fusion peptides derived from fusion proteins to induce fusion by themselves. Several sequences of the viral glycoproteins E1 and E2 of HCV were, for example, identified to be able to induce fusion, which are reviewed here.

## Introduction

**FIGURE 1:**
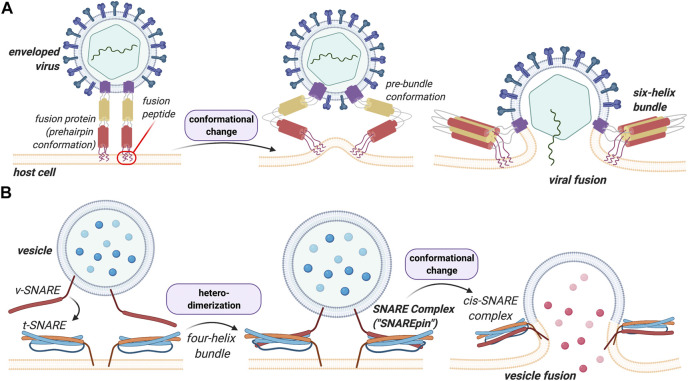
Structural and functional comparison between viral HA and eukaryotic SNARE in membrane fusion events. A key difference between these processes is that heterodimerization of the SNAREs is a prerequisite for the vesicular process whereas viral fusion proteins insert and destabilize the lipid bilayers to induce fusion in a more general manner. **(A)** Viral fusion events are mediated by the binding of fusion proteins to the membrane of the host cell. The proteins undergo a conformational change that allows the membranes to merge. **(B)** *v*-SNARE on the vesicle and the *t*-SNARE on the target membrane bind to one another leading to the formation of the *trans*-SNARE complex. A more detailed description of this process and its structural features can be found below.

Virus-cell fusion is the means by which enveloped viruses, including devastating human pathogens, bring their membrane into contact with a host-cell membrane such that their genetic contents can enter the host cells and initiate genomic replication. This process is mediated by one or more glycoproteins on the virus surface of which one is generally considered the fusion protein. These proteins drive the fusion of the two interacting membranes by undergoing a major conformational change that is triggered by interactions with the target cell (Figure 1A). While not common, this process also exists in eukaryotes where it has been implicated in a number of specific pathologic and biological processes, most notably in fertilization, but also in phagocytosis, pinocytosis, vesicular trafficking and the release of neurotransmitters from nervous synapses (White, 1992; Galli et al., 2002; Paumet, 2003; Dimitrov, 2004; Lorin et al., 2007; Meher and Chakraborty, 2019). In eukaryotes, intracellular membrane fusion is mostly mediated by soluble *N*-ethylmaleimide-sensitive factor attachment receptor (SNARE) proteins, whose association allows the membrane fusion process to occur (Figure 1). Eukaryotic SNARE proteins exist in two distinct forms—the vesicular, *v*-SNARE, and the target, *t*-SNARE, form—that specifically pair to one another leading to the formation of the SNARE complex (also called *trans*-SNARE, Figure 1B). The complex overcomes the energy barrier required for the fusion process (described below), and brings the two membranes close enough in space to cause sufficient distortion and stress to induce the bilayers to fuse (Galli et al., 2002; Witze and Rothman, 2002; Hofmann et al., 2004). Readers interested in the energetic aspects of the fusion process are directed to the work of Manca and coworkers on the subject (Manca et al., 2019).

In viruses and in contrast to eukaryotes, fusion is mediated by one or more surface proteins. In the case of the influenza virus, for instance, this protein is hemagglutinin (HA), for which the fusion mechanism has been extensively studied and characterized (Yang et al., 2015). Both HA and SNARE proteins share considerable structural similarity. The difference between the two fusion processes mediated by these proteins, however, is that heterodimerization of SNAREs proteins (*v*-SNARE with *t*-SNARE) is a prerequisite for fusion to occur, whereas HA, and viral fusion proteins more generally, insert and destabilize the lipid bilayer to induce fusion. This forms the basis of the present discussion.

In the interest of space, we have opted not to include a structural and mechanical discussion of the EFF-1 system, but would recommend that those who are interested consult the work of Zeev-Ben-Mordehai and co-workers on this topic (Zeev-Ben-Mordehai et al., 2014).

The intervention of viral fusion proteins, which, for the enveloped viruses, are essential for the infection step are of absolute importance, since the fusion of viral and host membranes is not a spontaneous process. Viruses can be enveloped or non-enveloped, and each has its own distinct mechanism of membrane fusion (Yamauchi and Helenius, 2013). With regard to the latter, the structural and functional properties of zwitterion-like fusion peptides have been described; these feature among the smallest fusion peptides that are currently known and are encoded by avian and Nelson Bay reoviruses (Shmulevitz et al., 2004).

In recent years, the structural characterization of various fusion proteins has allowed the crucial steps of this complex, multistage process to be elucidated (Yamauchi and Helenius, 2013; White and Whittaker, 2016). This has generally been achieved by a combination of electron cryomicroscopy, nuclear magnetic resonance spectroscopy (NMR), X-ray crystallography; their crystal structures, before and after the conformational rearrangement induced by the fusion, provide key information for our molecular understanding of the fusion mechanisms at play in these processes (Li and Modis, 2014).

**FIGURE 2:**
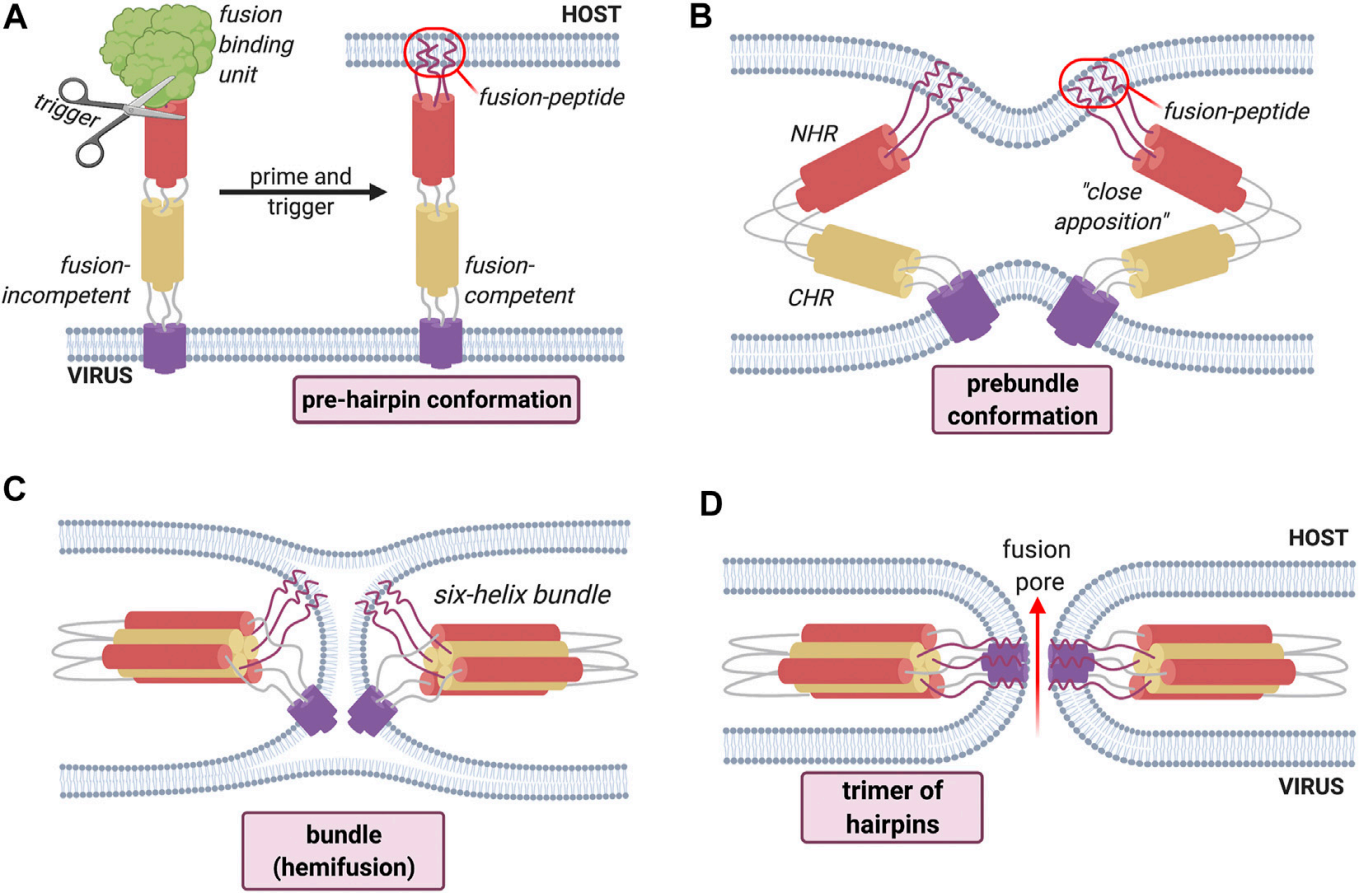
General mechanism of fusion process employed by Class I fusion proteins. An environmental trigger such as an acidic pH or the binding to a coreceptor (represented figuratively by the scissors) induces a conformational change that exposes the fusion peptide **(A)**. The fusion peptide then inserts into the host cell membrane causing the fusion proteins to fold back on themselves, inducing the bending of apposed membranes **(B)**. The folding creates a contact between the membranes, leading first to hemifusion **(C)**. Finally, the refolding leads to the formation of the fusion pore **(D)** and subsequent mixing of contents.

Turning to the manner in which fusion proteins exert their effects, it is known that they exist on the mature viral surface in a ‘native’ fusion-competent state. A generalized mechanism of fusion by class I fusion proteins can be described (Figure 2).

The “activation” of these proteins, in which the electrostatically repulsive barrier between the two membranes is overcome in order to achieve an active and functional (“*fusion competent*”) conformation, requires an environmental trigger which can be either pH-dependent or pH-independent, and is represented schematically by the scissors in Figure 2. These environmental triggers bring about a series of conformational changes that convert the native fusion-competent protein (i.e., the conformation in which it exists before any fusion-determining interaction with a host-cell membrane) into a membrane-embedded prehairpin that then allows the insertion of a key hydrophobic segment of a fusion protein into the membrane; this key segment is the fusion peptide itself. The fusion protein exists on the mature viral surface in a “native” fusion-competent state, which is most often, but not always, metastable, i.e., held in a conformation with one possible free energy minimum.

This insertion results in the formation of a trimeric conformation. The resulting conformation is a *prehairpin intermediate*, which embeds in the target membrane through the *fusion peptide.* This is followed by a sequence of conformational changes, during which the fusion protein folds back leading to the bending of the membranes 2). This will create a new point of contact between the membranes where only the outer bilayer leaflets fuse together; this step is called *hemifusion* 3). The last step is the fusion of the distal bilayer leaflet leading to the formation of a fusion pore allowing the mixing of the cellular contents 4).

All viral fusion proteins are heavily glycosylated and anchored into the viral envelope (Yamauchi and Helenius, 2013). They are displayed on the surface of enveloped viruses and can be classified according to key structural features of their pre- and post-fusion structures, which are often heavily conserved among members of the same virus family (Dimitrov, 2004). Until the 2000s, only two classes of viral fusion proteins (*i.e.,* Class I and Class II) had been identified. A third class (Class III) was later added and more recent studies point to the purported existence of additional classes, as numerous viral fusion proteins do not correspond to either of the three “classical” classes (Lorin et al., 2007; White et al., 2008; Falanga et al., 2009; Galdiero et al., 2012; Sobhy, 2017; Meher and Chakraborty, 2019).

**FIGURE 3:**
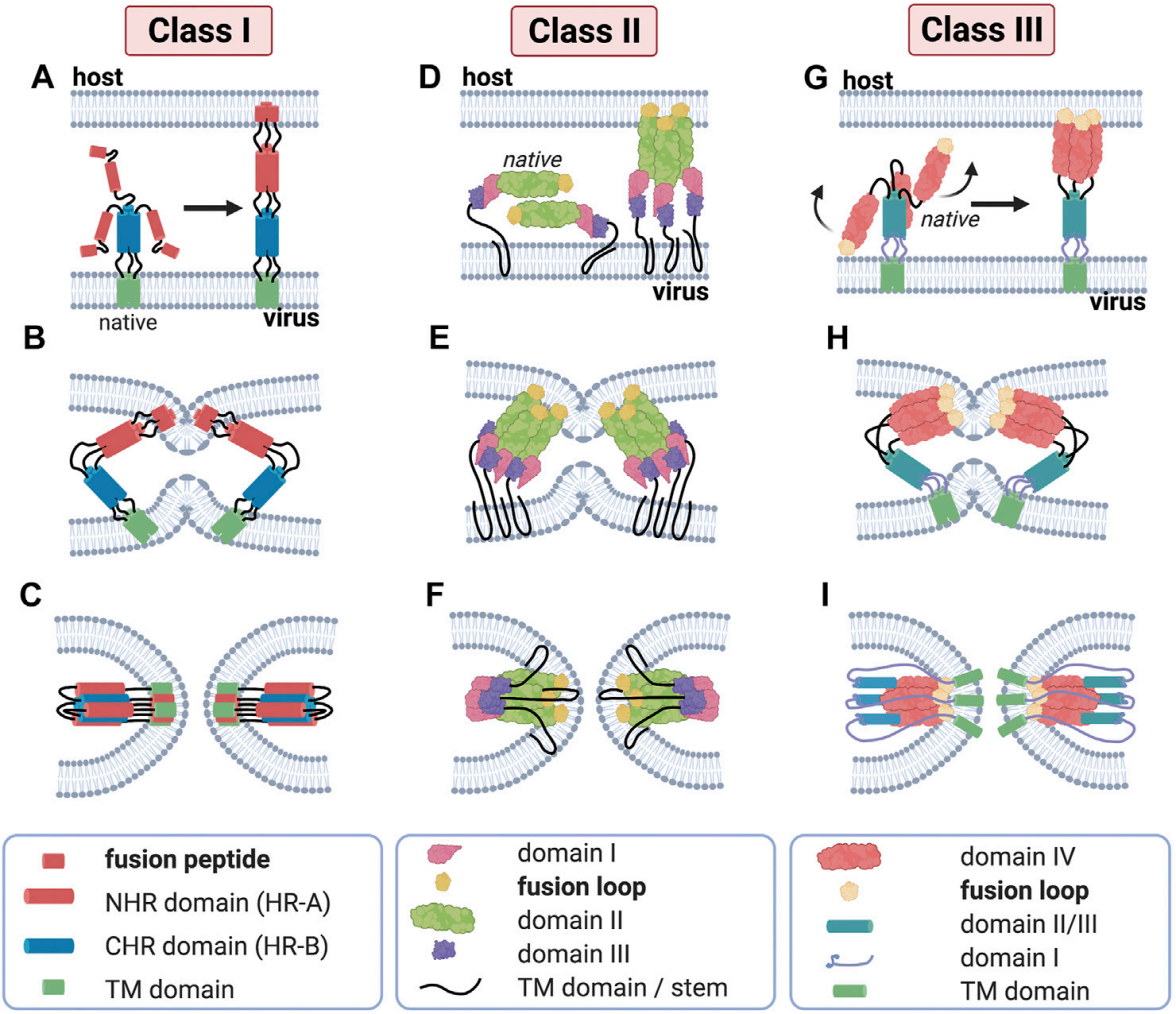
Major structural features of membrane fusion processes across the three canonical classes of fusion protein. (Column 1) Membrane fusion driven by viral class I proteins. The model of class I protein-mediated hemifusion and fusion depicts the progression through an extended prehairpin followed by breaking the threefold symmetry and dissociation of the *C*-heptad repeat domains. In the native prefusion conformation, the paramyxovirus fusion (F) protein consists of globular head domain attached to the transmembrane (TM) domains and short luminal tails through the TM domain-proximal heptad repeat sequences. When fusion commences, major structural rearrangements lead to assembly of the head-domain HR segments into a central, trimeric alpha-helical coiled-coil structure, displacing the fusion peptides in the direction of the host-cell membrane **(A)**. Subsequent hairpin-like refolding **(B)** then positions the heptad repeat domains into the grooves of the central triple helix, resulting in the formation of the stable six-helix bundle (6HB) post-fusion structure **(C)**, in which the fusion peptide-proximal core coiled-coil structure is surrounded by the three TMD-proximal HR helices; this is a defining feature of fusion proteins of this class. (Column 2) Class II viral protein-mediated fusion. Homo-/hetero-di-/trimers of E1/E2 **(D)** engage a target membrane with their fusion loops following low pH-induced conformational changes. Subsequent conformational changes involve the reorientation of domain III around a hinge region **(E)** that positions the TM region and the fusion loop (yellow) proximal to one another. Subsequent refolding of the extended trimeric conformation into a hairpin structure promotes hemifusion and fusion pore formation **(F)**. Despite their marked structural differences, proteins of class I and class II are able to progress through a similar refolding pathway. (Column 3) Membrane merger induced by class III viral fusogens. The native trimer of class III fusion proteins folds into a tripod-like arrangement, on which the fusion loops are positioned at the tip of each leg and are therefore directed into the viral envelope. Low pH conditions result in the protonation of a specific cluster of histidine residues that exert a major destabilizing effect on the prefusion structure. Once triggered, the tripod legs are proposed to swing upward **(G)**, driving the fusion loops toward the target membrane. Repositioning of the domains with conserved tertiary structure relative to each other through secondary structure reorganization in hinge regions and major changes of the trimerization domain **(H)** then ultimately result in a classic hairpin post-fusion conformation **(I)**.

**Class I** fusion proteins are present in *orthomyxoviruses*, *filoviruses*, *paramyxoviruses*, retroviruses and c*oronaviruses*. In this subclass, influenza virus and human immunodeficiency virus type 1 (HIV-1) are well studied and commonly used as models. The fusion peptide of this class—which is proteolytically released—is located at the post-proteolytic *N*-terminus of the glycoprotein (Dimitrov, 2004; Cross et al., 2009; Millet and Whittaker, 2018). The pre-fusion conformation is composed of a core of three bundled α-helices and which then undergo refolding into a 6-helix-bundle (6-HB) structure in the post fusion conformation (Figure 3). The class I membrane-fusion reaction is mediated by the refolding of the fusion protein to a highly stable rod-like structure with a central trimeric α-helical coiled coil.

The **class II** fusion proteins, which are to be found in the Flaviviridae and Togaviridae families, have a three-dimensional structure that is considerably different from that of the influenza virus HA, a finding that led to them being introduced as a separate class of fusion proteins (Lescar et al., 2001). Class II fusion proteins have a three-domain architecture composed to a large extent of β-strands with a tightly folded fusion loop in the central domain. Unlike class I fusion proteins, they are not oriented in a perpendicular fashion to the viral membrane but rather in a parallel or nearly parallel manner. Moreover, they are not proteolytically cleaved and do not form coiled coils (Galdiero et al., 2012). Class II proteins fold as homo- or heterodimers with another viral glycoprotein (Figure 3), as is the case for E1 proteins of togaviruses such as Semliki Forest virus (Tscherne et al., 2006). A common property of fusion proteins of Flaviviridae is that they are present at the viral surface as dimers. Another important difference between class I and class II fusion proteins is that class II fusion peptides, unlike members of class I, are not located in the *N*-terminal or *N*-proximal region but is buried in the dimer and forms a loop connecting β-strands at the tip of domain II. The fusion loop is composed mostly of apolar and other conserved residues, and the substitution of negatively charged amino acids in this crucial region can block fusion altogether (Allison et al., 2001). Monoclonal antibodies specifically targeting this region have revealed that this fusion loop inserts into membranes during fusion (Ahn et al., 2002; Gibbons et al., 2004).

The **class III** fusion proteins are employed by *herpesviruses, rhabdoviruses* and *baculoviruses*. This class features characteristics of both class I and class II fusion proteins. For example, they have a helical and a central β-stranded domain bearing one or more fusion loops. This is the case of rhabdoviral G proteins, which contrast class I fusion proteins in that they do not complex with other proteins on the virion surface. Like Class II fusion proteins, they are not proteolytically cleaved and do not feature heptad repeat sequences which are predictive of coiled-coils (Dimitrov, 2004). In its native form, rhabdoviral G protein is found as a homotrimer at the viral membrane folded into a tripod-like arrangement (Figure 3) (Roche, 2006; Roche et al., 2007). Upon environmental triggers, such as acidic pH, the trimer undergoes a major refolding, orienting the tripod legs towards cellular membrane and giving an extended prehairpin conformation where the fusion peptide is inserted in the host cell membrane. Similarly to class I and II fusion proteins, the prehairpin then folds back to an hairpin conformation juxtaposing the fusion peptide and transmembrane domain, leading to membrane merging (Plemper and Melikyan, 2013).

As pointed out above, some studies suggest that there are potentially further classes of fusion proteins, since some viruses, such as HCV or bovine viral diarrhea virus (BVDV), possess fusion proteins whose structures do not correspond to either of the three classes so far described (Falanga et al., 2009; Spadaccini et al., 2010; Moustafa et al., 2019).

The different virus families make use of different triggering mechanisms—including a pH-dependent and pH-independent manner (such as the interaction of the virus with a host-cell receptor)—to induce conformational change of the viral fusion protein which in turn, is essential for exposing the buried fusion peptide (Falanga et al., 2009; Yamauchi and Helenius, 2013; Li and Modis, 2014).

## Identification of Fusion Sequences

### General Concepts

Fusion peptides are constituent parts of the viral fusion proteins—specifically the sequence exposed during the fusion process—and are usually 20–30 residues in length. In the non-fusogenic state of the fusion protein, the fusion peptide is buried and protected in a hydrophobic crease of the soluble ectodomain (Li et al., 2003). The fusion peptide is exposed by the effects of an environmental trigger, thereby allowing it to insert into the host membrane. As a result, they are important for the fusion process since their anchoring to the host membrane is crucial for the connection of the two membranes (Millet and Whittaker, 2018).

The locations of the fusion peptide within the viral fusion proteins vary depending of the class fusion protein. Class I fusion proteins usually have a fusion peptide located at the *N*-terminus, while class II fusion proteins have an internal fusion loop and class III fusion proteins have bipartite fusion loops (Modis, 2013). In addition, fusion peptides can, be characterized by a number of different criteria, such as their hydrophobicity, resulting from their amino acid composition, or their flexibility, which is also a major feature of those peptides as the fusion process imparts a significant conformational rearrangement on the fusion proteins. However, it has been suggested that the fusion process is the concerted action of several discrete fusogenic sequences (Lavillette et al., 2007; Li et al., 2009; Galdiero et al., 2015).

### Fusion Abilities: *In Vitro* Assays

Fusion peptides perform a key role in the fusion process as they can interact directly with membranes. Hence, the determination of which exact segments play this role helps to unravel the steps of the fusion process. The identification of the candidate fusion peptides within whole protein sequences is mainly done according to the hydrophobicity and on the degree of conservation (Hernandez et al., 1996). After identifying potential fusion peptides, their fusion abilities are subsequently studied using different *in vitro* assays.

The first of these experiments determines the aggregation ability of the candidate sequences, since fusion proteins are supposed to cluster at the membrane during the fusion process. It has been proposed that fusion peptides can self-associate in the membrane and that this might assist in the recruitment of several fusion proteins into a single fusion site (Nieva and Agirre, 2003). The aggregation of vesicles can also be an important process to observe as it is one of the first steps of the membrane fusion process which implies two closely positioned membranes. The fusion peptides should be able to bring two membranes close enough for the membrane fusion to occur (Rapaport and Shai, 1994; Nieva and Agirre, 2003); it is therefore important to take into account the evaluation of membrane-membrane interactions in presence or absence of active peptides (Estes et al., 2006; Pacheco et al., 2006).

**FIGURE 4:**
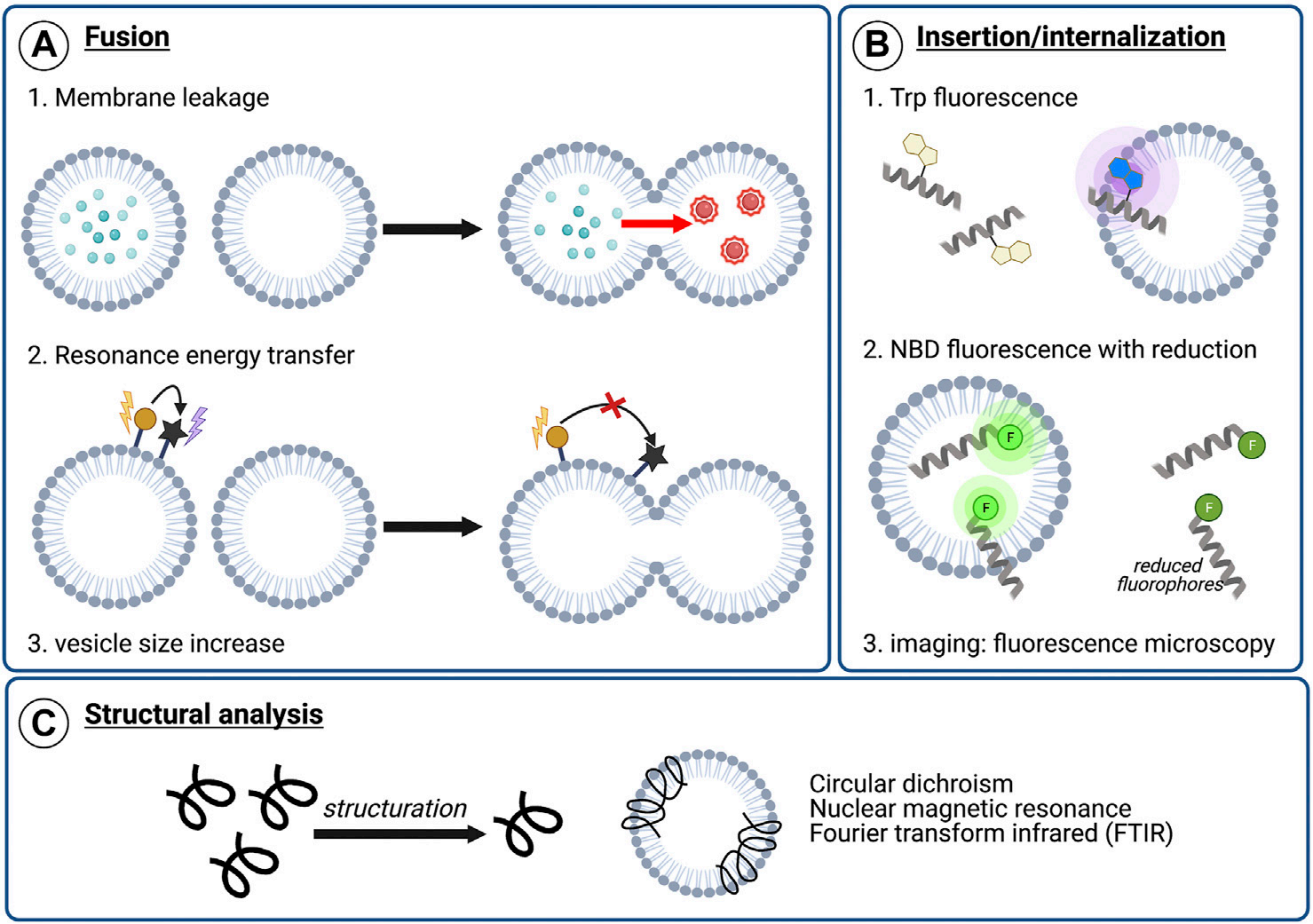
Examples of analytical techniques to characterize peptide’s membranotropic properties. **(A)** Fusogenic properties: 1) Membrane leakage experiments. After pore formation upon peptide addition, dilution of the self-quenching dye from labeled vesicles to unlabeled vesicles results in fluorescence increase, 2) Lipid mixing with FRET. The increasing distance between two fluorophores composing a FRET system upon lipid mixing can be visualized by monitoring the energy transfer efficiency decrease, 3) Lipid mixing can also be highlighted by dynamic light scattering (DLS) monitoring the vesicle size increase, **(B)** Insertion or internalization propensity: 1) Peptide insertion can be monitored by following Trp fluorescence. Upon membrane insertion, the hydrophobic environment around Trp results in a blue-shift in fluorescence, 2) NBD/sodium dithionite experiments. NBD-labeled sequences are incubated with liposomes and a reducing agent, sodium dithionite, is added. After reduction, the remaining fluorescence indicate the degree of membrane insertion/internalization, 3) Imaging experiments using fluorescence microscopy, such as confocal or total internal reflection fluorescent (TIRF) microscopy, with labeled peptide and liposomes or cells **(C)** Structural characterization in contact with model or cellular membranes to visualize conformational changes upon membrane interaction.

As fusion peptides represent a specific form of membranotropic peptides, they ought to induce perturbations in the lipid membrane. Nonetheless, it should be noted that this perturbation alone is not sufficient to determine whether fusion can be induced (Pérez-Berná et al., 2006). A second possible assay to highlight potential fusogenic properties is the leakage of vesicle contents (Figure 4A). Here, liposomes containing a fluorescent probe such as 5-carboxyfluorescein or calcein are mixed with unaltered vesicles (Weinstein et al., 1977). Since calcein undergoes self-quenching at high concentrations, formation of fusion pores upon fusogenic peptide addition should lead to an increase in fluorescence due to calcein leakage and dilution. Such signal increase could therefore confirm whether the peptide is able to form pores and potentially initiate fusion. Membrane leakage experiments have notably been successfully used with other techniques to identify membrane-active regions of proteins C and E from the dengue virus (Nemésio et al., 2011), the E1 and E2 glycoproteins of HCV (Pérez-Berná et al., 2006), or even of designed synthetic sequences (Wang et al., 2016). Lipid mixing is another important step involved in the fusion process and can be monitored by spectrofluorescence using Förster resonance energy transfer (FRET) probes, as was first described by Struck *et al.* (Struck et al., 1981). In such experiments, liposomes are marked with two different fluorescent probes in a FRET system. Upon mixing of unmarked vesicles and the fusion peptide, the resulting membrane fusion causes lipid mixing and thus dilution of the fluorescent probes within the membrane, leading, in turn, to a decrease of the resonance energy transfer efficiency. By directly measuring the fluorescence emission, it is possible to monitor the fusogenic activity of the peptide (Buzón et al., 2005; Taylor et al., 2009). Finally, vesicle size increase can be monitored to validate the lipid mixing. It can be measured by using dynamic light scattering which determines the diameter and size distribution of the vesicles (Buzón et al., 2005; Reichert et al., 2007).

Among membranotropic properties, membrane insertion is a crucial feature for many peptides. In order to characterize the membrane insertion propensity of tryptophan-containing sequences, Trp fluorescence can be monitored (Figure 4B). Indeed, since fluorescence is dependent on the environment of the chromophore, peptide insertion will lead to a blue-shift of the Trp emission wavelength due to a more hydrophobic environment. As a result, measuring Trp fluorescence represents a simple and easily-accessible technique for characterizing membrane insertion, and it was used, for instance, in experiments studying a HA2 influenza fusion peptide analogue (Zhelev et al., 2001) or the ebola fusion peptide (Freitas et al., 2007).

The internalization of the peptides can also be monitored by spectrofluorescent spectroscopy using the nitrobenzoxadiazole (NBD)/dithionite assay (Gonzalez et al., 2019). In this technique, the membranotropic peptide is conjugated to a fluorescent probe, NDB, whose fluorescence property can be turned off *via* chemical reduction using sodium dithionite (McIntyre and Sleight, 1991). Hence, liposomes and the fluorescently marked peptide are mixed in order to allow membrane activity. Since the intravesicular medium is not accessible to the reducing agent, the decrease in fluorescence after chemical reduction could only be related to peptides in solution. Thus, the remaining fluorescence should indicate the degree of membrane insertion and/or internalization of the peptides in vesicles. This technique was efficiently used to characterize the activity of various membranotropic peptides such as SynB peptide vectors (Drin et al., 2003), penetratin and analogues (Terrone et al., 2003), several cell-penetrating peptides (CPPs) and antimicrobial peptides (AMPs) (Swiecicki et al., 2014) as well as HCV E1 and E2 (Gonzalez et al., 2019). Alongside these, fluorescence microscopy—especially with confocal or total internal reflection fluorescent (TIRF) microscopes—can also be used to determine whether a marked peptide was able to insert or internalize into model membranes or in biological membranes (Otterstrom and van Oijen, 2013; Gonzalez et al., 2019).

Since conformational structuration upon membrane interaction is a crucial feature for fusion activity, structural information of peptides in solution and in contact with model membranes such as micelles or liposomes represent key information. Such structural studies (Figure 4C) of these peptides can be performed by circular dichroism (CD), nuclear magnetic resonance (NMR), fluorescence spectroscopy or Fourier transform infrared (FTIR) spectroscopy, in the presence or absence of micelles or liposomes to identify residues inserted, the insertion depth and whether conformational changes were or have been induced (Freitas et al., 2007; Scrima et al., 2014). Atomic force microscopy (AFM) has also been used to investigate peptide-lipid membrane interactions, but a worthy description of the calculations required for this are beyond the scope of this review, but are elegantly described by Utjesanovic and co-workers (Utjesanovic et al., 2019).

Such methods and shared characteristics among the investigated peptides (*vide infra*) allowed the definition of various fusion peptides (Table 1). The following peptides were identified according to the methods described in this section. It can be noted that similarities exist between fusion peptides within the same family such as conservations of residues’ motives or of their nature (aromatics, small residues, *etc.*). The nature of these sequences is described in more detail below.

**TABLE 1:** Examples of fusion peptides from different virus families.

Fusion class	Family	Virus	Fusion Peptide	Fusion protein	Refs.	
Class I	Retroviridae	HIV-1	AVGIGALFLGFLGAAGSTMGARS	gp41	Lorin et al. (2007); Galdiero et al. (2012); Apellániz et al. (2014); Falanga et al. (2018)	
		FIV	AAIHVMLALATVLSIAGAGTGATA	gp36	Lorin et al. (2007)	
		HTLV-1	AVPVAVWLVSALAMGAGVAGGITGS	gp21	Apellániz et al. (2014)	
		SIV	GVFVLGFLGFLATAGSAMGAAS	gp32	Crowet et al. (2012); Apellániz et al. (2014)	
		ASLV	GPTARIFASILAPGVAAAQALREIERLA	EnvA	Apellániz et al. (2014)	
		BLV	SPVAALTLGLALSVGLTGINVAVS	gp30	Apellániz et al. (2014)	
	Orthomyxoviridae	Influenza A	GLFGAIAGFIENGWEGMIDG	HA	Lorin et al. (2007); Galdiero et al. (2012); Apellániz et al. (2014)	
		Influenza B	GFFGAIAGFIEGGWEGMIAGHGY	HA	Lorin et al. (2007)	
	Filoviridae	Ebola	GAAIGLAWIPYFGPAAEGIYTEGL	GP2	Freitas et al. (2007); Galdiero et al. (2012); Apellániz et al. (2014); Falanga et al. (2018)	
		MARV	LAAGLSWIPFFGPGI	GP2	Apellániz et al. (2014)	
	Paramyxoviridae	Sendai	FAGVIGTIALGVATSAQITAGIA	F1	Lorin et al. (2007); Li et al. (2009); Galdiero et al. (2012)	
		SV5	FAGVVIGLAALGVATAAQVTAAVA	F1	Lorin et al. (2007); Li et al. (2009)	
		SV41	VSANQAGSRRKRFAGVVVGLAALGVATAAQ	F1	Lorin et al. (2007); Li et al. (2009)	
		Measles	FAGVVLAGAALGVATAAQITAGIA	F1	Lorin et al. (2007); Li et al. (2009)	
		HeV	LAGVVMAGIAIGIATAAQITAGV	F	Apellániz et al. (2014)	
		NDV	FIGAIIGSVALGVATAAQITAA	F	Apellániz et al. (2014)	
		PIV5	FAGVVIGLAALGVATAAQVTAAVALV	F	Apellániz et al. (2014)	
	Coronaviridae	SARS-CoV	MYKTPTLKYFGGFNFSQILPDPFL (*N*-ter)	S	Lorin et al. (2007); Apellániz et al. (2014)	
			GAALQIPFAMQMAYRF (internal)					
		hCoV	AFSLANVTSFGDYNLSSVLPQRNI	S2	Lorin et al. (2007)	
Class II	Togaviridae	SFV	VYTGVYPFMWGGAYCFCDS	E1	Galdiero et al. (2012); Apellániz et al. (2014)	
		CHIKV	VYPFMWGGAYCFCDTENT	E1	Apellániz et al. (2014)	
	Flaviviridae	DNV	DRGWGNGCGLFGKGSL	E	Li et al. (2009); Galdiero et al. (2012); Apellániz et al. (2014); Falanga et al. (2018)	
		WNE	VDRGWGNGCGLFGKGSIDTCAKFACSTKAIGR	E	Li et al. (2009)	
		JE	TDRGWGNGCGLFGKGSIDTCAKFSCTSKAIGR	E	Li et al. (2009)	
		YF	SDRGWGNGCGLFGKGSIVACAKFTCAKSMSLF	E	Li et al. (2009)	
		SLE	VDRGWGNGCGLFGKGSIDTCAKFTCKNKATGK	E	Li et al. (2009)	
		TBEV	CGLFGKGSIVACVKAAC	E	Garry and Dash (2003); Li et al. (2009)	
Class III	Herpesviridae	HSV	WFGHRY	gB	Galdiero et al. (2012); Apellániz et al. (2014); Falanga et al. (2018)	
			VEAF					
	Rhabdoviridae	VSV	WY	G	Apellániz et al. (2014); Falanga et al. (2018)	
			YA					
	Baculoviridae	AcMNPV	YAYNGGSLDPNTRV	gp64	Apellániz et al. (2014); Falanga et al. (2018)	
			VKRQNNNHFAHHTCNK					

### Shared Characteristics of Fusion Peptides

#### Hydrophobic Peptides and Methods to Identify Fusion Peptides

One of the main characteristics of fusion peptides is their hydrophobicity and, as such, they are usually composed of a high number of alanine (Ala) and aromatic residues but also glycine (Gly) is prevalent (Lavillette et al., 2007; Galdiero et al., 2012, 2015; Apellániz et al., 2014). It was noted that ‘small residues’ (*i.e.* Ala, Gly) were not found in other hydrophobic segments of the fusion proteins (Nieva and Agirre, 2003; Lorin et al., 2007). The side chains of aromatic residues interact with lipids of the target cell and help to stabilize the peptide in a region of the lipid raft close to interface of the two membranes (Epand, 2003). Tryptophan (Trp) is the most common aromatic amino acid present in this kind of sequence and, in fact, its indole ring appears to anchor the peptide in micelles (Alves et al., 2016). In viruses such as HIV, influenza A and SIV, the glycine residues play an important role in the oligomerization of the fusion peptides and the necessary amphipathicity and orientation of the fusion peptides for the membrane fusion to occur, since one “face” of the helix would be richer in Gly (Lorin et al., 2007). The Gly residues may have a role in forming the required structure of the fusion peptide to allow the destabilization of the membrane. Furthermore, the study of paramyxoviral fusion peptides show that the Gly residues may be involved in the regulation of the activation of the fusion protein (Russell et al., 2004).

Given that hydrophobicity is one of the main characteristics of these sequences, several groups have used the Wimley-White interfacial hydrophobicity scale (WWIHS)—an experimentally-determined free energy scale that measures the propensity of individual amino acids in unfolded peptide chains to partition from water to the lipid bilayer interface—to identify the most hydrophobic regions of viral fusion proteins and to calculate the overall interfacial hydrophobicity of a particular peptide sequence (Pacheco et al., 2006; Lavillette et al., 2007; Taylor et al., 2009; Garry et al., 2011; Badani et al., 2014; Galdiero et al., 2015). The WWHIS is experimentally derived from measurements of the free energy of amino acids transfer at the interface (Phoenix et al., 2002). By using the WWIHS, it is possible to determine the hydrophobicity of sequences upon examination of the partitioning of residues into electrostatically neutral interfaces, showing, for example, that sequences containing aromatic amino acids are more likely to interact at the lipid-bilayer interface (Wimley and White, 1996). As an example of its application in practice, Basso and coworkers used WWIHS to spatially determine SARS-CoV fusion peptides within the fusion protein, spike (S), and were subsequently able to identify two membranotropic regions (770–778) (MWKTPTLKYFGGFNFSQIL) and (873–888) (GAALQIPFAMQMAYRF. Table 2). Their findings were confirmed by vesicle membrane leakage studies, indicating their role in the fusion process. It was then suggested that the fusion protein SARS-CoV S2 (a subunit of S) is organized such that the putative fusion peptide (770–778) is then followed sequentially by an internal fusion peptide (873–888) (Guillén et al., 2005, Guillén et al., 2008a, Guillén et al., 2008b; Millet and Whittaker, 2018). Alternatively, the interfacial helical hydrophobic moment (iHHM) is a physico-chemical factor that considers the membrane interaction and the secondary structure formation of peptides bound to membranes. When a peptide folds into an α-helix, the iHHM determines the degree of separation of the hydrophobic and hydrophilic faces and as a consequence, and can then help to identify peptides potentially able to form amphipathic helices (Galdiero et al., 2015). Other hydrophobic scales have also been used such as the Kyte-Doolittle scale, which can be used to pinpoint hydrophobic regions in proteins as well as to identify surface-exposed and transmembrane regions (Table 2) (Kyte and Doolittle, 1982). The Eisenberg hydrophobicity scale is another example that has been used to identify a conserved hydrophobic region of E2 [residues (495–515)] of HCV, the so-called the tridentate region (Table 2) (Eisenberg et al., 1984; Taylor et al., 2009). This region is able to insert into the lipid bilayer in a similar way as the peptide loops of SFV and tick-borne encephalitis virus (TBEV).

**TABLE 2:** Peptides identified with fusogenic properties.

Method	Peptide identified	Refs.
WWHIS	SARS-CoV fusion peptide and internal fusion peptide [770–788] MWKTPTLKYFGGFNFSQIL [873–888] GAALQIPFAMQMAYRF	Guillén et al., 2008b
Kyte Doolittle scale and WWHIS	Fusogenic peptides of HCV [430–449] NDSLYTGWLAGLFYHHKFNS [543–560] RPPLGNWFGCTWMNSTGF.[603–624] ITPRCLVNYPYRLWHYPCTINY	Pacheco et al. (2006)
Einsenberg hydrophobicity scale	Tridentate region of HCV [495–515] PYCWHYPPRPCGIVPAKSVCGPVYCFTPSPVV	Taylor et al. (2009)
Recurrence Quantification Analysis	HCV [259–298] RRHIDLLVGSATLCSALYVGDLCGSVFLVGQLFTFSPRHH [352–383] HWGVLAGIKYFSMVGNWAKVLVVLLLFAGVDA	Bruni et al. (2009)

Similar hydrophobicities of two segments within a fusion protein may imply a possible interaction between them. This can be illustrated by the work on the fusion peptide and the *C*-terminal region of the glycoprotein E1 of HCV (Table 2). By means of a coevolution method and Recurrence Quantification Analysis (RQA), these peptides have been identified as having a common hydrophobicity pattern and a capacity to interact in the post-fusion conformation, suggesting an interaction between these domains during the fusion process (Bruni et al., 2009).

#### Conservation of Residues

Fusion peptides may be different between the different virus families but within families sequences are usually well conserved (Galdiero et al., 2012; Millet and Whittaker, 2018). The amino acid sequence homology between any two given viral fusion proteins is usually less than 20% but when the two fusion peptides are derived from proteins within the same virus family the similarities can rise to as much as 90% (Nieva and Agirre, 2003). For instance, fusion peptides from the glycoprotein E (Flaviviridae family) show great similarities (Table 1). This can also be evidenced with the well-studied viruses HIV and influenza. The alignment of fusion sequences from all types (HIV-1, HIV-2 and SIV; influenza A and influenza B) show well-conserved residues and motifs, such as GFLG in retroviruses (Durell et al., 1997; Lorin et al., 2007).

Aside from the identification of the hydrophobic amino acids (*i.e.,* the “*small*’ and aromatic residues taken together) characteristic of the fusion peptides, sequence alignment of fusion proteins can be useful to identify the fusion peptide candidates and several groups have identified motifs conserved in different families of viruses. For instance, the class II fusion peptides have conserved motifs such as DRGWGNGCGLFGKG for flaviviruses or GVYPFMWGGAYCFCDSEN for alphaviruses (Lavillette et al., 2007). The motif I^800^EDLLF^805^ of the fusion peptide of SARS-CoV was found to be highly conserved within the Coronaviridae family (Millet and Whittaker, 2018). Furthermore, recent research found 96% similarity in the structure and in the amino acid composition of SARS-CoV two compared to SARS-CoV, which first appeared in 2002/2003 in China (Sternberg et al., 2020; Tang et al., 2020). It was also recently shown by Tang and coworkers that the motif (SFIEDLLFNKV) was strongly conserved among the fusion peptides of SARS-CoV, MERS-CoV and SARS-CoV 2 (Tang et al., 2020).

Additionally, it can also be note that not only are the residues conserved but also the type of residues (e.g., aromatic, polar) (Durell et al., 1997). Conserved patterns shared by fusion peptides from fusion proteins which lack an overall sequence homology could be linked to a conservation of function. In fact, mutations of conserved amino acids in a sequence can lead to a loss of activity (Epand, 2003; Drummer et al., 2007; Taylor et al., 2009). Mutagenesis plays a key role in examining the function of regions presenting a fusion potential, for instance the sequence 276–286 of HCV contains class II fusion peptide-like characteristics (*e.g.,* the VFLVG motif and conserved cysteines) (Nieva and Agirre, 2003; Drummer et al., 2007). For the GFLG motif in retroviruses, mutations diminished or abolished fusogenic properties of the peptide, suggesting its key role in the fusion process (Lorin et al., 2007).

Lavillette *et al.* studied three hydrophobic and conserved regions of HCV. They proceeded by aligning sequences from different HCV genotypes and subtypes. Their aim was to study the influence of the substitution of conserved and non-conserved residues and the results show an influence of the conserved residues for the folding of the protein that occurs during the fusion process. Modifications of non-conserved residues, however, had no influence. A loss of activity in mutant peptides is suggestive of their implication and importance in the fusion process (Lavillette et al., 2007).

For instance, the tridentate region is conserved in all HCV variants, as well as in Flaviviridae and Togaviridae (Taylor et al., 2009). The peptide was tested by liposome fusion assays followed by FRET, to measure its fusogenic capacities, showing similar lipid mixing as with the fusion peptide of SFV. Mutations of Gly or aromatic residues located in the middle of the peptide rendered the peptide non-infectious.

Coevolution studies can also help to provide information about viral protein functions and conformational changes that can guide antiviral strategies, as amino acid coevolution corresponds to mutations of different residues within a similar timeframe. The HCV fusion mechanism has been investigated using this method, revealing a region of E2 co-evolving with amino acids of E1. The E1 residues—and a region of E2 called *back layer* - rely on interaction with each other in order to undergo a folding rearrangement for the fusion peptide insertion (Douam et al., 2018). The coevolution method was also investigated by Bruni *et al.* that identified cross-recurrence for FP (259–298) and a *C*-terminal anchor region (331–383) of E1 of HCV in a dataset of five genotypes of HCV, meaning that an interaction could be involved in the post-fusion conformation of the glycoprotein. Since the crystal structure of the post-fusion conformation cannot be obtained, the existence of such interactions could provide insight into the conformation of the glycoprotein (Bruni et al., 2009). Thus, the interaction between the FP and the *C*-terminal anchor of E1 implicate their role in the first step of the membrane fusion leading to hemifusion. This study, mentioned in *N-Terminal Region of E1 (197–214)*, may show that not only is the composition of amino acids conserved, but also the hydrophobicity.

#### Structure

Fusion peptides often have a unique structure, about which there is sometimes a certain degree of controversy (Buzón et al., 2005; Reichert et al., 2007). The overall conformation depends heavily on the conditions of the structural experiments, such as the conformation of a segment of the viral fusion protein as a crystal in the absence of membranes, or the study of small synthetic fusion peptides in presence or absence of membranes (Epand, 2003).

The structure of the fusion peptides is dependent on the lipid composition and the polarity of the environment in which they are studied. Different structural analyses, such as NMR, CD or IR, show a multitude of different structures of the fusion peptides and their synthetic analogues when the lipid environment is varied. The peptide-to-lipid ratio, itself, can also have an influence on the conformation of the fusion peptides (Martin et al., 1994; Dimitrov, 2004; Freitas et al., 2007; Lorin et al., 2007; Scrima et al., 2014; Galdiero et al., 2015; Meher and Chakraborty, 2019). The fusion peptide of the influenza virus adopts β-sheet-like structures in DMSO, whereas in trifluoroethanol and aqueous buffer, a mixture of both α and β structures could be observed (Lüneberg et al., 1995). Martin and coworkers demonstrated that their synthetic peptides underwent a conformational transition to α-helical structures in a lipid environment, compared to peptides dissolved in DMSO (Martin et al., 1994). These conformational changes were described as a consequence of the peptides’ capacity to interact with membranes, as shown for fusion peptide candidates of HCV, which were studied to be used as cell-penetrating agents. Their conformations were determined by CD or NMR in presence of water or lipid-mimetic environments. The authors’ conformational results showed an increase in the extent of ordered structure, suggestive of an interaction of the peptides with the micelles (Gonzalez et al., 2019).

Results such as these lead to contradictory conclusions about the structure of the fusion peptides, particularly in the case of HIV fusion peptide, gp41, which either adopts an α-helical conformation or β-sheet structure according to the peptide-to-lipid ratios and lipid compositions. As a consequence, studies have been conducted to elucidate this matter. Búzon *et al.* studied and compared kinetic information of the two most widely used fusion peptides of HIV under the same conditions, leading to the conclusion that lipid mixing depends on the transformation of unordered and helical structures into aggregated β-structures and that such a conformational change occurs upon binding to the membrane (Buzón et al., 2005). With the same intention of elucidating whether either conformation is relevant for the fusion process, Reichert *et al.* used a different approach by incorporating l- and d-CF_3_-phenylglycine in various positions of the fusion peptide from gp41. The incorporation of the two enantiomers would confirm the hypothesis of α-helical peptides and β-stranded oligomers. The l-CF_3_-phenylglycine should not influence the oligomerization or the helical structuration. But the d-CF_3_-phenylglycine, because of the steric obstruction, would impair the oligomer formation and helical folding. Even though the l- or d-epimers were able to induce different conformations, no conformational *preference* was observed during the fusion assays, suggesting that for this peptide no particular structuration was needed (Reichert et al., 2007).

**FIGURE 5:**
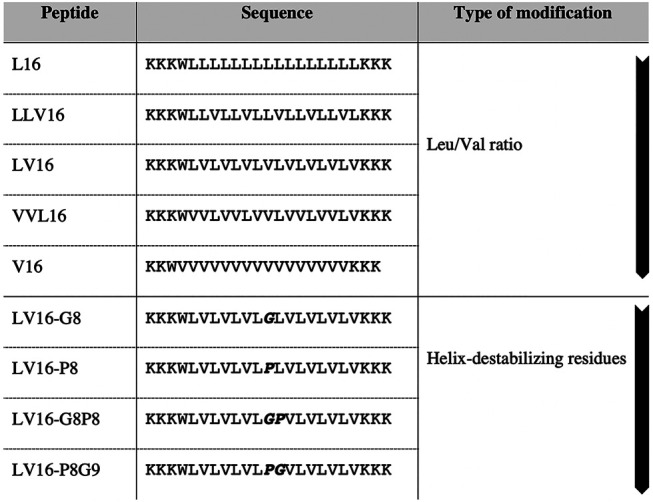
Design of fusogenic peptides containing different ratios of helix promoting, β-sheet-promoting or helix destabilizing residues (in bold) adapted from Hofmann *et al.* The first arrow indicates the increase of Val ratio. The second arrow indicates the increase of helix destabilization (Hofmann et al., 2004).

Hence, the environmentally-determined conformational changes imply a significant degree of flexibility inherent in this class of hydrophobic peptides (Meher and Chakraborty, 2019). As mentioned above, the peptides have a high content of Ala/Gly, which is hypothesized to result in their flexibility (Drummer et al., 2007; Li and Modis, 2014). To illustrate this point, a study conducted by Hofmann *et al.*, in which α-helix-promoting Leu and Val residues were incorporated in peptides at different ratios. They observed that the fusogenicity of these peptides was dependent on the ratio of these residues and that the fusogenic properties were also enhanced by incorporation of helix-destabilizing Pro and Gly residues within their hydrophobic cores (Figure 5). These modifications induced a large degree of structural flexibility and showed up to 80-fold increased fusogenicity for the active peptides (Hofmann et al., 2004).

As previously mentioned, aromatic residues also play an important role in these sequences and in the structuration of the peptide. The point-mutation of aromatic residues in the Ebola fusion peptide destabilized the helical structure (Freitas et al., 2007). Scrima and coworkers studied C6a, C6b and C8; short peptides derived from gp36 of FIV. (Scrima et al., 2014). These peptides possess a motif featuring a regularly spaced Trp, which is essential for the turn-shaped backbone conformation due to the supposed orientation of the indolyl rings.

The selected conformers were superimposed at level of the backbone heavy atoms showing 0.32 Å and 0.47 Å RMSD for C6a and C6b, respectively. Analysis of the backbone dihedral angles showed the prevalence of C6a to adopt regular β-turn structures encompassing the residues W773-W776 which are stabilized by hydrogen-bonding between the carbonyl of 772D and the HN of 775G and 776W. NMR analyses showed that the Trp indolyl rings in C6a adopt a parallel orientation that allow a large hydrophobic surface to be exposed.

Another structure frequently observed in a number of different fusion peptides is the so-called *tilted* fusion peptide. Angle insertion, the angle of the peptide compared to the membrane surface, has been studied and linked to the fusogenic capacity of those peptides but was still in debate in 2007 (Lorin et al., 2007). This angle is influenced by different factors such as the lipid composition or peptide length (Apellániz et al., 2014). The oblique orientation of the fusion peptides is linked with the hydrophobicity of the peptide which is asymmetrically distributed along the helix according to the Brasseur model, which delineates the interaction between hydrophobic peptides and lipid bilayers. Accordingly, the most hydrophobic end penetrates more deeply in the core of the membrane whereas the other end will have more affinity with polar heads of the membrane (Lins et al., 2006; Lorin et al., 2007). Several examples have shown the importance of the orientation of the peptide during the insertion into lipid environments. A fusion peptide of the influenza virus (GLFGAIGFIEGGWTGMIDG), located in the HA2 subunit, has been shown to insert into hydrophobic environments at an oblique angle independent of the pH of the surroundings (Lüneberg et al., 1995). The oblique insertion of fusion peptides was also predicted computationally and observed experimentally for other viruses. For instance, the SIV fusion peptide (GVFVLGFLGFLA), located on the *N*-terminus of the glycoprotein gp32, was studied by Martin *et al.*, who concluded that the oblique angle of insertion was linked to the fusogenic activity (Martin et al., 1994). This suggests not only that hydrophobicity and the capacity to destabilize lipids are sufficient for fusion, but also that the oblique insertion of the fusion peptide may also be important. This peptide has been further studied by Bradshaw *et al.* who, in turn, confirmed an oblique insertion at 55 by neutron diffraction (Bradshaw et al., 2000). By means of computational studies (from Coarse-Grained to Atomistic Model) Crowet and coworkers established a significant correlation with previous experimental studies of the SIV fusion peptide (Crowet et al., 2012).

### Conclusions

The criteria described above are useful for identifying fusion peptides, given that they are usually hydrophobic and well conserved within a particular family of viruses. Some doubt remains around the structure of these peptides with some authors taking the view that they adopt α-helical structures but given the conformational dependence of the fusion peptides on the nature of their environment, they should be considered flexible. It should also be pointed out that structural analysis of the fusion peptide within a protein and a synthetic peptide is not the same. For instance, Murata *et al.* modified the *N*-terminus of a fusogenic sequence of HA protein of influenza virus (Murata et al., 1991), which brought about a loss of activity despite the similar secondary structures of both the native and the modified peptide, as determined by CD spectroscopy in the presence of small unilamellar vesicles at acidic pH. These results show that α-helix formation alone is not sufficient to trigger membrane fusion and critical amino acids should be taken into account.

Hence, it should be noted that a single criterion is not sufficient to determine the fusogenic property of the peptide. Studies by Serrano *et al.* determined a relationship between the structure and activity of the HIV-1 fusion peptide. While maintaining the hydrophobicity of the peptide, they modified the amino acid composition of the conserved motif LFLGFLG. The change of order led to a different fusion peptide secondary structure. It also modified the interactions of the peptides at the membrane surface and prevents the oligomerization for the formation of the trimer (Serrano et al., 2017). As a result, a deeper understanding of the structural requirements for the fusion peptide interaction and fusogenic activity towards membranes would be useful for understanding the mechanism and to guide further studies towards, as an example, drug delivery applications. In addition, the analysis of those characteristics would provide clues about the structure and function of fusion peptides. These peptides could also serve as representative models in studies of viral fusion processes because of the similarities between the results with the fusion proteins, as has been suggested by Nieva *et al* (Nieva and Agirre, 2003).

## Case Study: Hepatitis C Virus

### Setting

According to the WHO, 71 million people worldwide were living with HCV in 2015 (World Health Organization and Global Hepatitis Programme, 2017). Approximately 70–80% of those infected with HCV develop chronic hepatitis, in a further 20–30% of whom, the disease progresses to liver cirrhosis (Triyatni et al., 2002; Alves et al., 2016). As a result, HCV is still a major burden that has to be considered by healthcare systems around the world, as the actual treatment has a high cost. Early treatments against HCV consisted of various types of interferon which were later replaced by the small-molecule treatment ribavirin, which was approved in 1999. But patients using this treatment suffered from poor responses and development of severe adverse effects including depression or anxiety. The market approval of direct-acting antivirals (DAAs), such as sofosbuvir in 2013, which could directly inhibit the replication cycle of HCV, led to an improvement for HCV therapy. However, access to HCV therapy remains limited. Among those diagnosed in 2015, only 7.4% started a direct-acting antiviral treatment (World Health Organization and Global Hepatitis Programme, 2017).

HCV, itself, is an enveloped virus of the Flaviviridae family discovered in 1988. Its genome is composed of a single stranded RNA (ssRNA) encoding a polyprotein that is broken down during infection into structural proteins (C, E1 and E2) and non-structural proteins (NS2, NS3, NS4A, NS4B, NS5A and NS5B) (Moustafa et al., 2019).

**FIGURE 6:**
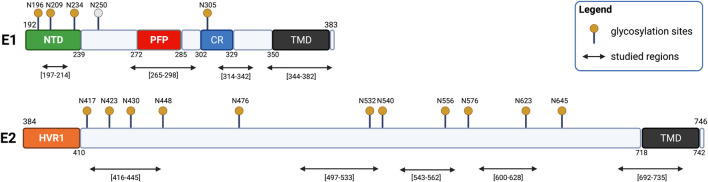
Schematic representation of HCV glycoproteins E1 and E2. E1 is composed of an *N*-terminal domain (NTD, yellow), a putative fusion peptide (PFP, red), a conserved region (CR, blue), and a transmembrane domain (TMD, black). E2 is composed of two hypervariable regions (HVR1 and HVR2, orange) and a transmembrane region (TMD, black). The glycosylation site N250 is specific to genotype 1b/6.

The fusion process of HCV is mediated by two transmembrane glycoproteins: E1 and E2 (with residues 192–383 and 384–746, respectively) (Pacheco et al., 2006; Drummer et al., 2007; Yin et al., 2017). Bioinformatic analyses revealed a conserved organization of the glycoprotein E1 composed of the *N*-terminal domain (192–239), a putative fusion peptide (272–285), a conserved region (302–329) and the transmembrane domain (350–381) (Figure 6) (Tong et al., 2018). A conserved organization is also observed in glycoprotein E2 composed of a hypervariable region HVR1 (384–410) and the transmembrane domain (710–746) (Figure 6) (Lavillette et al., 2007). The E2 glycoprotein was previously suggested to be a class II fusion protein as well as E1, though without the receptor-binding function (Alves et al., 2016).

HCV possesses two envelope glycoproteins E1 and E2 that each have their own transmembrane anchors. Some doubt remains about the structure of the glycoproteins E1 and E2, as crystal structures of the glycoproteins were only partially resolved, with structural data only for E1 *N*-terminal region (PDB code 4UOI) (El Omari et al., 2014) and E2 hydrophobic core domain (PDB code 4MWF) being available (Kong et al., 2013).

Initially, E2 was the most studied of both proteins and was thought to be the archetypal fusion protein of HCV (Pacheco et al., 2006; Tong et al., 2018). However, the putative HCV fusion peptide is now thought to be located in E1, (Alves et al., 2016), with suggestions that the E1 glycoprotein acts as the fusogenic subunit while E2 acts as the chaperone and mediates receptor binding (Alves et al., 2016; Nosrati et al., 2017). One of the arguments in favor of this view is the capacity of E1 to form a trimer, a typical structural feature of all fusion proteins (cf. Figure 3) (Tong et al., 2018). However, it should be noted that the membranotropic properties of other regions within E1 and E2 have since been studied and identified. This suggests that the fusion process could involve a cooperative action of both E1 and E2 and consequently, of the concerted actions of membranotropic peptides (Epand, 2003; Pérez-Berná et al., 2006; Lavillette et al., 2007). HCV fusion seems therefore to be mediated by the non-covalently linked E1-E2 heterodimer (Triyatni et al., 2002; Tong et al., 2018; Colpitts et al., 2020). Recently, a combination of electron microscopy, coevolution theory, homology and already-established partial protein structures led to modeling of full length E1E2 heterodimer in pre-fusion conformation (Castelli et al., 2017; Freedman et al., 2017). Gopal and colleagues have, nevertheless, also described the cooperative role of E1 and E2 (Gopal et al., 2017). In such constructs, the putative HCV fusion peptide, located in E1, seems to have a partial helical structure and is supposedly in interaction with the E2 hypervariable region 2 (HVR2), thus reinforcing the hypothesis of its chaperone role (Cao et al., 2019).

Even though a putative fusion peptide (272–285) has been identified for HCV, several other membranotropic sequences are described in the literature (Table 3). In the following subsections, we will present sequences, within E1 and E2 HCV fusion proteins, able to interact with membrane and representing possible membranotropic peptides. They were selected based on their composition (sequence homology, mutagenesis), their properties (hydrophobicity, structure) and membrane activity. The sequences with their associated protein and residues, are presented in Table 3.

**TABLE 3:** Sequences within the E1 and E2 fusion proteins examined as part as possible membranotropic peptides in the current discussion.

Glycoprotein	Sequence	
**E1**	197–214	SSGLYHVTNDCPNSSVVY
	265–298	LVGSATLCSALYVGDLCGSVFLVGQLFTFSPRHH
	314–342	TGHRMAWNMMMNWSPTAALVVAQLLRIPQ
	344–382	IMDMIAGAHWGVLAGIKYFSMVGNWAKVLVVLLLFAGVD
**E2**	416–445	TNGSWHINSTALNCNESLNTGWLAGLFYQH
	497–533	VPAKSVCGPVYCFTPSPVVVGTTDRSGAPTYSWGAND
	543–562	RPPLGNWFGCTWMNSTGFTK
	600–628	GPRITPRCMVDYPYRLWHYPCTINYTIFK
	692–737	LHQNIVDVQYLYGVGSSIASWAIKWEYVVLLFLLLADARVCSCLWM

### Structure of E1 and E2 Glycoproteins

#### *N*-Terminal Region of E1 (197–214)

The E1 *N*-terminal region was studied by both Garry *et al.* and Garcia *et al.* for its inhibitory effects on entry and for its binding to HepG2 cells (Garcia et al., 2002; Garry et al., 2011). This peptide comprises residues (197–214) (SSGLYHVTNDCPNSSVVY) of E1 corresponds to the *N*-terminal region of E1. When aligning the sequence across different genotypes we can observe a conservation of its residues (Table 4) (The UniProt Consortium, 2021).

**TABLE 4:** Sequence alignment of region 197–214 across various genotypes. In bold are represented the highly conserved residues.

Genotype	Sequence
HCV1a (H77)	S**S**GL**Y**HV**TNDC**P**N**S**S**V**V**Y
HCV1b (BK)	V**S**GI**Y**HV**TNDC**S**N**A**S**I**V**Y
HCV2a (BEBE1)	T**S**SS**Y**MA**TNDC**S**N**S**S**I**V**W
HCV5a (SA13)	A**S**GV**Y**HV**TNDC**P**N**S**S**I**V**Y
HCV3a (NZL1)	T**S**GL**Y**VL**TNDC**S**N**S**S**I**V**Y
HCV6a (6a33)	S**S**GL**Y**HL**TNDC**P**N**S**S**I**V**L
HCV4a (ED43)	V**S**GI**Y**HV**TNDC**P**N**S**S**I**V**Y

Within this region, Garcia *et al.* reported that the sequence (192–211) YQVRNSTGLYHVTNDCPNSS could potentially bind to HepG2 cells. The partially overlapping peptides such as (202–221) HVTNDCPNSSIVYEAADAIL, however, were unable to bind to the HepG2 cells, implying that the *N*-terminal peptide identified by the authors is essential for hepatocyte-binding activity (Garcia et al., 2002).

More recently, the structure of the E1 *N*-terminal region in solution was obtained and described by El Omari *et al.* In this study, the authors showed that this region adopts an unexpected structure and forms a covalently linked homodimer which does not correspond to a truncated class II fusion protein folding, as originally hypothesized for E1 protein (El Omari et al., 2014).

To our knowledge, despite its known hydrophobicity (Garry et al., 2011), no studies demonstrated the potency of this region as a membranotropic sequence or its implication in the membrane fusion process. Moreover, its capacity to bind to hepatocytes or to form covalent dimers could indicate a role more dedicated to recognition or protein crosstalk.

#### Putative Fusion Peptide: Residues (265–298)

This sequence (LVGSATLCSALYVGDLCGSVFLVGQLFTFSPRHH) located in E1 ectodomain, was identified by several groups and is considered as the putative fusion peptide of HCV, showing the various characteristics of fusion peptides (Pérez-Berná et al., 2006; Lavillette et al., 2007; Tong et al., 2018). This sequence has one of the strongest membranotropic effects among E1 and E2 glycoprotein-derived sequences (Pérez-Berná et al., 2006).

The (265–298) region is thought to be a truncated class II fusion peptide as it has small homology to the class II fusion peptides of flavivirus glycoprotein E (Drummer et al., 2007) but it still has some shared features such as the VFLVG motif and a total of three conserved Cys. Generally, the Cys residues are thought to be involved in disulfide bonds, but it was shown by Li *et al.* (Li et al., 2009) that C272 and C281 are more likely to be directly involved in the fusion mechanism as they were unlikely to form disulfide bond. Furthermore, their results show that the alanine mutations of the two cysteines inhibited the fusion cell capacity. Another conserved sequence, G^278^DLC^281^ was identified by Tong *et al.* as significant since the mutation of those residues leaded to the inhibition of fusion (Tong et al., 2017).

Additionally, the region is highly conserved as shown when aligning the sequence with all the genotypes of HCV (Table 5). It was subsequently compared to other fusion peptides of flavivirus and paramyxovirus showing the peptides’ conserved nature (Garry and Dash, 2003; Li et al., 2009; Moustafa et al., 2019). In this sequence, the conserved residues are Gly267, Cys272, Gly278, Asp279, Cys281, and Gly288. Mutagenesis studies allowed the identification of a relationship between the reduction in membrane fusion and the conserved residues. Mutations C272A, C281A, G282A and G288A lead to the inhibition of cell fusion by 18–51% (Li et al., 2009). In addition, Lavillette *et al.* studied the modification of Y276F and G282A impairing the entry capacity (Lavillette et al., 2007).

**TABLE 5:** Sequence alignment of the region (265–298) across various HCV genotypes (Li et al., 2009). In bold are represented the highly conserved residues. In green, amino acids modified by mutagenesis. Underlined conserved motives GDLC and VFLVG.

Genotype	Sequence
HCV1a (H77)	LVGS**A**TL**CS**AL**Y**V**GD** L **CG**SVFLVG **Q**LFTFS**P**RH**H**
HCV1b (BK)	LVGA**A**AF**CS**AM**Y**V**GD** L **CG**SVFLVS**Q**LFTFS**P**RR**H**
HCV2a (BEBE1)	IVMS**A**TL**CS**AL**Y**V**GD**V**CG**ALMIAA**Q**VVVVS**P**QH**H**
HCV5a (SA13)	LAGG**A**AL**CS**AL**Y**V**GD**A**CG**AVFLVG **Q**MFTYS**P**RR**H**
HCV3a (NZL1)	LVGA**A**TM**CS**AL**Y**V**GD**M**CG**AVFLVG**Q**AFTFR**P**RR**H**
HCV6a (6a33)	LAGA**A**VV**CS**SL**Y**I**GD** L **CG**SLFLAG**Q**LFTFQ**P**RR**H**
HCV4a (ED43)	MVGA**A**TV**CS**GL**Y**I**GD** L **CG**GLFLVG**Q**MFSFR**P**RR**H**

The putative region was studied by the synthesis of a truncated chimeric protein, as described by both Lombana *et al.* and Tong *et al.* (Lombana et al., 2016; Tong et al., 2017). The protein is lacking the residues (268–292), which did not modify the overall structure. Both the native and chimeric proteins showed destabilization of the membrane, but in the case of the chimeric protein, a 15-fold higher concentration was needed for the membrane leakage test (Lombana et al., 2016). In both studies, a decrease of infectivity was observed when comparing the protein lacking the putative region and the native protein. Finally, a short peptide (YVGDLSGSVFL) of this region has also shown fusogenic property as determined by experiments described by Gonzalez *et al.* as well as Struck *et al.* (cf. *Fusion Abilities: In Vitro Assays*) (Struck et al., 1981; Gonzalez et al., 2019). In these conditions different structures could be observed in different environments by CD. The peptide adopts a random coil conformation in water, but adopts β-structures in presence of micelles (Gonzalez et al., 2019).

#### E1 Protein Region (314–342)

This E1 region (TGHRMAWNMMMNWSPTAALVVAQLLRIPQ), corresponding to the protein pre-transmembrane domain (pre-TMD) appears to have a role in the fusion process. It was studied by Spadaccini and colleagues (Spadaccini et al., 2010). By aligning the sequences from a number of HCV genotypes, as shown in Table 6, the authors noticed that this region is highly conserved and was predicted to form a transmembrane helix.

**TABLE 6:** Alignment of segment 314–342 from various genotypes of HCV (Spadaccini et al., 2010). In bold are represented the highly conserved residues. In red and blue are the α-helices (319–323) and (329–338) respectively.

Genotype	Sequence
HCV1a (H77)	T**GHRMAW**N**MMM**N**WSP**TAALVVAQLL**R**I**P**Q
HCV1b (BK)	S**GHRMAW**D**MMM**N**WSP**TTALVVSQLL**R**I**P**Q
HCV2a (BEBE1)	T**GHRMAW**D**MMM**N**WSP**TTTMLLAYLV**R**I**P**E
HCV5a (SA13)	T**GHRMAW**D**MMM**N**WSP**TTALVMAQLL**R**I**P**Q
HCV3a (NZL1)	S**GHRMAW**D**MMM**N**WSP**AVGMVVAHVL**R**L**P**Q
HCV6a (6a33)	T**GHRMAW**D**MMM**S**WSP**TTTLVLSSIL**R**V**P**E
HCV4a (ED43)	T**GHRMAW**D**MMM**N**WSP**TTTLVLAQVM**R**I**P**T

This same region was designated in separate work by Pérez-Berná to be involved in the promotion of membrane destabilization, pore formation and enlargement in a similar way to pre-transmembrane and/or loop domains of class I fusion proteins (Pérez-Berná et al., 2006). The fusion and membrane fusion assays, showed high values for the segment (317–339) (Pérez-Berná et al., 2006).

**FIGURE 7:**
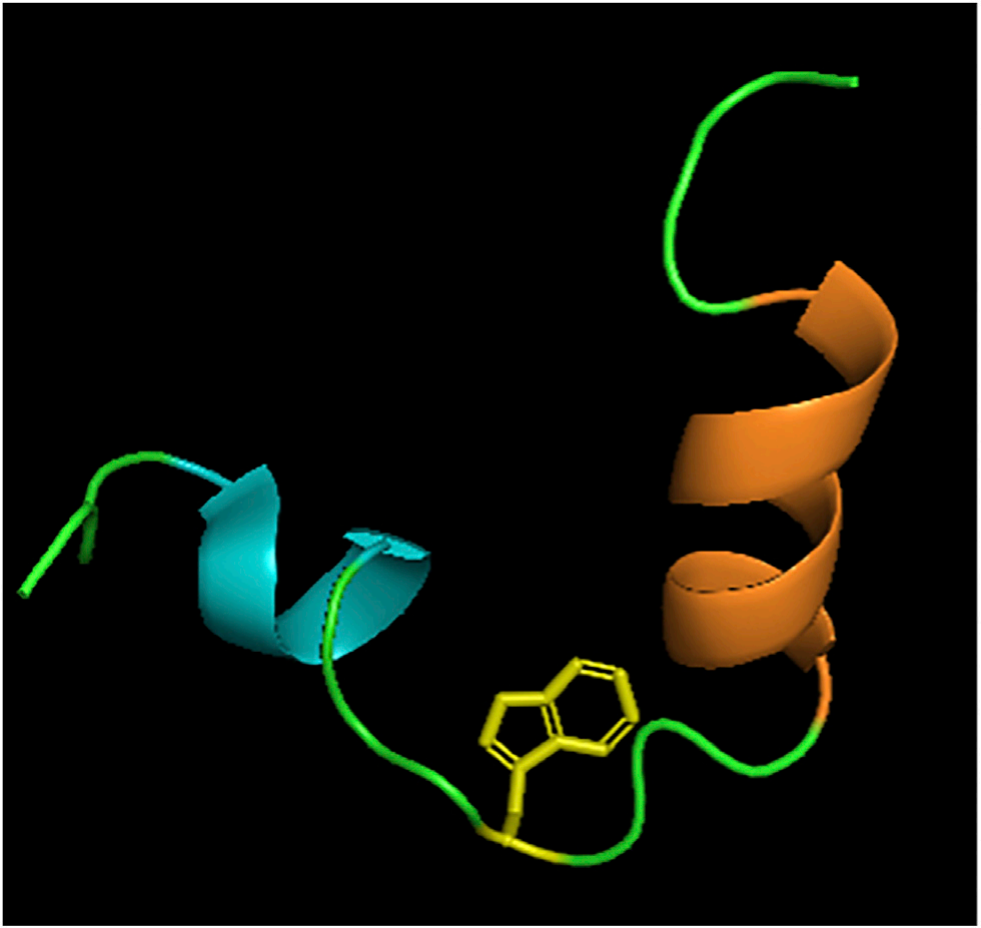
Structure of E1 pre-transmembrane region determined by NOE experiments (Spadaccini et al., 2010). Two alpha helices MAWDM (cyan) and AALVVAQLL (orange) separated by a bend illustrated by Trp326 (yellow). PDB code: 2KNU.

NOE and CD experiments in different media suggested a propensity for helical structures and that this region is adopting a boomerang structure of two helical stretches between residues (319–323) and (329–338), which is a shared feature with the influenza virus fusion peptide. These two helical stretches are interrupted by Trp326, at which point the peptide forms a bend (Figure 7) (Spadaccini et al., 2010). Its structure is stabilized by making contacts with the side chains of Trp326 and Pro328, and Trp326 and Ala331. In 2015, Kong and co-workers revealed the crystal structure of a cross-neutralizing antibody that recognizes the HCV E1 glycoprotein (Kong et al., 2015). In the crystal structure of the antibody in complex with the E1 glycopeptide, obtained at 1.75Å resolution, the team showed the antibody binds to one face of an α-helical peptide. At the same time, molecular dynamics suggested that E1 linear peptide, in the absence of ligand, is flexible in solution.

#### *C*-Terminal Region of E1 (344–382)

This region (IMDMIAGAHWGVLAGIKYFSMVGNWAKVLVVLLLFAGVD) of the E1 protein is located at the *C*-terminus of E1 and is encompassing the transmembrane domain (TMD) (Table 7 **)**. This region is located across both the transmembrane domain (353–383) and the *C-*terminal region of E1 (Bruni et al., 2009). The *C*-terminal region is composed of two sequences: an amphipathic pre-anchor domain (331–347)—described in *E1 Protein Region (314–342)*—and the transmembrane region (353–383).

**TABLE 7:** Alignment of segment (344–382) from various genotypes of HCV. In bold are represented the fully conserved residues. In red and blue are the pre-anchor domain and transmembrane region respectively.

Genotype	Sequence
HCV1a (H77)	IMDMIA**G**A**HWG**VLAGIK**Y**FSMVGN**W**A**KV**LVVLLLFA**GV**D
HCV1b (BK)	VVDMVA**G**A**HWG**VLAGLA**Y**YSMAGN**W**A**KV**LIVMLLFA**GV**D
HCV2a (BEBE1)	VLDIIT**G**G**HWG**VMFGLA**Y**FSMQGA**W**A**KV**VVILLLTA**GV**E
HCV5a (SA13)	VIDIIA**G**A**HWG**VLFAAA**Y**YASAAN**W**A**KV**VLVLFLFA**GV**D
HCV3a (NZL1)	LFDIMA**G**A**HWG**ILAGLA**Y**YSMQGN**W**A**KV**AIIMVMFS**GV**D
HCV6a (6a33)	CASVIF**G**G**HWG**ILLAVA**Y**FGMAGN**W**L**KV**LAVLFLFA**GV**E
HCV4a (ED43)	LVDLLS**G**G**HWG**VLVGVA**Y**FSMQAN**W**A**KV**ILVLFLFA**GV**D

The sequence (360–382) appears to play a role in the fusion process according to Pérez-Berná and coworkers, who were able to identify it thanks to its positive hydrophobicity score and interfaciality and its hemifusion and fusion values (Pérez-Berná et al., 2006). Bruni *et al.* showed that the *C*-terminal region interacts with the putative fusion peptide (259–298) in the post-fusion structure by using two computational approaches; the first based on the coevolution theory and the second on *Recurrence Quantification Analysis* (which allows a direct comparison of domains of common hydrophobicity patterns) (Bruni et al., 2009). This relationship between the two segments may be part of a glycoprotein refolding mechanism during the fusion process.

#### E2 Protein Region (416–445)

The peptide sequence (416–445) (TNGSWHINSTALNCNESLNTGWLAGLFYQH) of E2 protein has been identified as one of the membranotropic regions of the E2 glycoprotein and is highly hydrophobic (Alves et al., 2016). Lavillette *et al.* identified a peptide, spanning residues (416–430), that matches the criteria of hydrophobicity, conservation and residue composition (Lavillette et al., 2007). In this same region, the sequence (430–449) was identified by the Wimley-White hydrophobicity plot to have a high propensity for membrane partition, especially the sequence of residues G^436^WLAGLFYQ^444^ (Pacheco et al., 2006; Alves et al., 2016). The presence of the characteristic residues (small residues and aromatic) represents 45% of the sequence, which is a common feature of fusion peptides (Table 8). The presence of the GLF motif can also be considered a defining trait, as it is shared and conserved among class I fusion proteins (Alves et al., 2016).

**TABLE 8:** Alignment of segment 416–445 from various genotypes of HCV. In bold are represented the highly conserved residues. In green, amino acids modified by mutagenesis. Underlined, the GxxxG motif. In red, glycosylation sites.

Genotype	Sequence
HCV1a (H77)	TNG**SWHIN**S**TALNCN**E**S**LN**TG** WL **A** G **L**F**Y**QH
HCV1b (BK)	TNG**SWHIN**R**TALNCN**D**S**LQ**TG**FL**A**A**L**F**Y**TH
HCV2a (BEBE1)	TNG**SWHIN**R**TALNCN**D**S**LE**TG**FL**A**A**L**F**Y**TS
HCV5a (SA13)	TNG**SWHIN**R**TALNCN**D**S**LQ**TG** FV **A** G **L**L**Y**YH
HCV3a (NZL1)	TNG**SWHIN**S**TALNCN**E**S**IN**TG** FI **A** G **L**F**Y**YH
HCV6a (6a33)	NGS**SWHIN**R**TALNCN**D**S**LQ**TG**FL**A**S**L**F**Y**VR
HCV4a (ED43)	SNG**SWHIN**R**TALNCN**D**S**LN**TG**FL**A**S**L**F**Y**TH

In another study, reduced cell-cell fusion was observed for the mutation of G418A, G418D compared to wild-type E1E2, due to their role in membrane fusion as the binding to CD81 of the mutant proteins was not impaired (Lavillette et al., 2007). Alves *et al.* have shown that the peptide (421–445) can interact with micelles through the insertion of hydrophobic residues into the micellar membranes (Alves et al., 2016). A short peptide (419–432) from this region showed by spectrofluorescent spectroscopy using NBD/dithionite assay (*Fusion Abilities: In Vitro Assays*)*,* the capacity to interact with the membrane as the peptide could insert or even internalize into large unilamellar vesicles (LUVs) and cells with a better activity in an anionic environment (Gonzalez et al., 2019). The sequence (430–449) studied by Pacheco *et al.* showed a pH-dependent release of aqueous content from lipidic vesicles, reaching 100% content release at acidic pH and 10% of leakage at neutral pH (Pacheco et al., 2006). This could be linked to the presence of two His residues that could play a role as pH sensors, which is common feature among some flaviviruses (Alves et al., 2016).

Using CD spectra, Alves *et al.* observed that the sequence (427–436) is present as a random coil in solution but that conformational changes were induced by addition of detergent monomers. The peptide contains four Asn residues and one Gln, which can function as both hydrogen-bond donors and acceptors alongside their known contribution to helical stability. A second motif (**GxxxG**) present in the peptide is also known for its structural stability, as a consequence of the weak hydrogen bonds facilitated at the Gly position (Table 8) (Alves et al., 2016). In contrast, when Pacheco and coworkers analyzed a similar sequence (430–449) via FTIR spectroscopy in D_2_O medium buffer in absence of lipid, the sequence mainly showed extended β-structures (Pacheco et al., 2006). The structural difference observed may be due to the difference in analytical methods applied and the small differences in the sequences such as residue composition and length.

It was noted by Lavillette *et al.* that three glycosylation sites are present in this region at position 417, 423 and 430 which is not a usual feature of the fusion peptide and W420A mutation has an influence on the binding to CD81. Thus, despite its membranotropic properties, this region may not have a direct role in the fusion process (Lavillette et al., 2007).

#### E2 Protein Region (497–533)

The sequence (497–553) from E2 located in the protein’s hydrophobic core (VPAKSVCGPVYCFTPSPVVVGTTDRSGAPTYSWGAND) was also studied for its membrane activity. On account of the hydrophobicity of this region, the sequence (497–515) was detected by Pacheco *et al.* with the Kyte-Doolittle scale—but not with the Wimley-White scale—meaning that it is not predicted to induce membrane destabilization at the interface, but was suggested to interact with the internal region of the bilayer (Pacheco et al., 2006). A shorter sequence, studied by Taylor *et al.*, (495–515), located in the Tridentate region of HCV E2, was identified to have a conserved hydrophobicity profile among the different analyzed genotypes with the Einsenberg hydrophobicity profile (a normalized consensus hydrophobicity scale sharing many features with the other hydrophobicity scales) of E1 and E2 (Taylor et al., 2009). Beyond the conservation of its hydrophobicity, its sequence composition was also conserved among the HCV variants and other members of the Flaviviridae family (Table 9). This peptide was able to induce lipid membrane mixing in a similar way to the SFV fusion peptide while having a better fusogenic capacity when exposed to low pH (Taylor et al., 2009).

**TABLE 9:** Alignment of segment 497–533 from various genotypes of HCV. In bold are represented the fully conserved residues.

Genotype	Sequence
HCV1a (H77)	**VPA**KS**VCGPVYCFTPSPVV**V**GTTD**RS**G**A**PTY**S**WG**A**N**D
HCV1b (BK)	**VPA**SE**VCGPVYCFTPSPVV**V**GTTD**RF**G**V**PTY**R**WG**E**N**E
HCV2a (BEBE1)	**VPA**RT**VCGPVYCFTPSPVV**V**GTTD**RA**G**A**PTY**N**WG**E**N**E
HCV5a (SA13)	**VPA**RG**VCGPVYCFTPSPVV**V**GTTD**RK**G**N**PTY**S**WG**E**N**E
HCV3a (NZL1)	**VPA**SS**VCGPVYCFTPSPVV**V**GTTD**AR**G**V**PTY**T**WG**E**N**E
HCV6a (6a33)	**VPA**ST**VCGPVYCFTPSPVV**I**GTTD**RR**G**N**PTY**T**WG**E**N**E
HCV4a (ED43)	**VPA**SS**VCGPVYCFTPSPVV**V**GTTD**HV**G**V**PTY**T**WG**E**N**E

The E2 sequence (504–522) was suggested to be important for the fusion as it was identified as having strong membranotropic properties and mutations within the sequence that could suppress the fusion activity (Alves et al., 2016). The location of this sequence buried in the hydrophobic core of the E2 structured region, however, makes it unlikely to serve as a fusion peptide.

The sequence (513–533) studied by Garcia *et al.* also shows binding activity to HepG2 cells (Garcia et al., 2002). According to the Gene data bank this is the most conserved peptide among the peptides studied by the authors. The region (525–660) also overlaps with the sequence that is described below (section 4.3.7) and is involved in determining the correct folding and subunit aggregation, with segments of this region being implicated in CD81 binding (Pérez-Berná et al., 2006). Indeed, the region (523–535) encompasses critical and conserved amino acids for the binding to CD81 (Alves et al., 2016).

#### E2 Protein Region (543–562)

The (543–562) sequence (RPPLGNWFGCTWMNSTGFTK) is also located in the E2 hydrophobic core. Within this region, Pacheco and coworkers identified the sequence (543–560) as hydrophobic according to both the Wimley-White and Kyte-Doolittle scales (Pacheco et al., 2006). This sequence is also rich in Ala, Gly and Phe, which is a characteristic of the fusion peptides (Pacheco et al., 2006). Moreover, this sequence shows a high conservation among HCV genotypes (Table 10). The interfacial hydrophobicity plot revealed that the sequence has the capacity to destabilize membrane. It was then confirmed *via* release of aqueous content from vesicles, during fluorescence quenching assays, showing a leakage of 90%, independently on the pH (Pacheco et al., 2006). The structure of the peptide was analyzed by FTIR spectroscopy in D_2_O medium buffer, showing a majority of extended β-structures, and few changes could be observed when acidic phospholipids were added (Pacheco et al., 2006). The authors did not comment on the nature of these changes.

**TABLE 10:** Alignment of segment 543–562 from various genotypes of HCV. In bold are represented the highly conserved residues.

Genotype	Sequence
HCV1a (H77)	**RPP**L**G**N**WFGC**T**WMN**S**TGF**T**K**
HCV1b (BK)	**RPP**Q**G**N**WFGC**T**WMN**S**TGF**T**K**
HCV2a (BEBE1)	**RPP**K**G**A**WFGC**T**WMN**G**TGF**T**K**
HCV5a (SA13)	**RPP**T**G**N**WFGC**T**WMN**S**TGF**V**K**
HCV3a (NZL1)	**RPP**S**G**R**WFGC**S**WMN**S**TGF**L**K**
HCV6a (6a33)	**RPP**T**G**G**WFGC**T**WMN**S**TGF**T**K**
HCV4a (ED43)	**RPP**H**G**A**WFGC**V**WMN**S**TGF**T**K**

#### E2 Protein Region (600–628)

The (600–628) (GPRITPRCMVDYPYRLWHYPCTINYTIFK) region from E2 identified by Lavillette as one of the most hydrophobic regions of E2 (Lavillette et al., 2007). Sequence [603–624] is rich in aromatic residues (Table 11), which is a common pattern for pre-transmembrane regions in retroviruses and filoviruses (Pacheco et al., 2006). Furthermore, the sequence (601–623) was identified as one of the regions responsible for the fusion process, since mutation in this region prevented membrane fusion (G600D, G600A, W602A,W616A) (Lavillette et al., 2007).

**TABLE 11:** Alignment of segment 600–628 from various genotypes of HCV. In bold are represented the highly conserved residues. In green, amino acids modified by mutagenesis. In red, aromatic residues.

Genotype	Sequence
HCV1a (H77)	**GP**RI**TPRC**MVD**YPYRLWH**Y**PCT**INYTIFK
HCV1b (BK)	**GP**WL**TPRC**MVD**YPYRLWH**Y**PCT**VNFTIFK
HCV2a (BEBE1)	**GP**WL**TPRC**LVD**YPYRLWH**Y**PCT**VNYTIYK
HCV5a (SA13)	**GP**WV**TPRC**LVD**YPYRLWH**Y**PCT**VNFTVHK
HCV3a (NZL1)	**GP**WL**TPRC**MVD**YPYRLWH**Y**PCT**VDFRLFK
HCV6a (6a33)	**GP**WL**TPRC**LVH**YPYRLWH**Y**PCT**LNYTIFK
HCV4a (ED43)	**GP**WI**TPRC**LID**YPYRLWH**F**PCT**ANFSVFN

The release of aqueous content from vesicles observed by Pacheco *et al.* was able to reach 100% with a low effect of the pH, showing the capacity of the peptides to perturb membranes. To follow, lipid mixing assays were performed, confirming its capacity of fusion of 60% at neutral pH (Pacheco et al., 2006).

Pérez-Berná and colleagues determined that the sequence (603–635) was one of several membranotropic regions of E2 and that this region was implicated in folding and receptor binding (Pérez-Berná et al., 2006). Molecular dynamics (MD) simulations performed by Taylor *et al.* revealed that (602–624) has a high propensity to insert into the lipid bilayer and the authors noted that their experimental findings were in line with these simulations (Taylor et al., 2009). This sequence contains also two central Cys residues and a central Pro suggesting that it could adopt a loop structure which, in turn, is thought to insert into the lipid bilayer (Pacheco et al., 2006). As regards its shape, the structure was analyzed by FTIR spectroscopy, revealing mainly extended β-structures in D_2_O medium buffer (Pacheco et al., 2006).

However, this region may also play a role in the binding to CD81 receptor which is not a feature held by fusion peptides (Liu et al., 2016). There are some contradictions in the literature concerning the role of this sequence. As described in this section, this segment of E2 show membranotropic properties. Hence, the region (600–628) has an important role in the fusion process but as the fusion mechanism of HCV is this not well understood yet, and since this sequence is located in the hydrophobic core, it may imply that its role is not as a fusion peptide (Alves et al., 2016; Colpitts et al., 2020).

#### E2 Transmembrane Domain (692–737)

The final region to be discussed, (692–737) (LHQNIVDVQYLYGVGSSIASWAIKWEYVVLL FLLLADARVCSCLWM) is positioned at the *C*-terminal end of E2. The alignment of the different genotypes of HCV shows that this region is highly conserved and present the characteristics residues such as aromatics and small residues (Table 12).

**TABLE 12:** Alignment of segment 600–628 from various genotypes of HCV. In bold are represented the highly conserved residues. In red and blue the aromatic and small residues respectively.

Genotype	Sequence
HCV1a (H77)	**LHQNIVD**V**QYLYG**VGSSIASWAI**KWE**YVV**L**L**FL**L**LADAR**VCSC**LW**M
HCV1b (BK)	**LHQNIVD**V**QYLYG**IGSAVVSFAI**KWE**YVL**L**L**FL**L**LADAR**VCAC**LW**M
HCV2a (BEBE1)	**LHQNIVD**V**QYLYG**LSPAITKYVV**KWE**WVV**L**L**FL**L**LADAR**VCAC**LW**M
HCV5a (SA13)	**LHQNIVD**T**QYLYG**LSSSIVSWAV**KWE**YIV**L**A**FL**L**LADAR**ICTC**LW**I
HCV3a (NZL1)	**LHQNIVD**V**QYLYG**VGSGMVGWAL**KWE**FVI**L**V**FL**L**LADAR**VCVA**LW**L
HCV6a (6a33)	**LHQNIVD**V**QYLYG**VSSSVTSWVV**KWE**YIV**L**M**FL**V**LADAR**ICTC**LW**L
HCV4a (ED43)	**LHQNIVD**V**QYLYG**VGSAVVSWAL**KWE**YVV**L**A**FL**L**LADAR**VSAY**LW**M

A region at the *C*-terminal of E2 shows also membrane destabilization properties. Pérez-Berná *et al.* were able to identify this as 713–737 for its hemifusion and fusion properties and the region (702–745) for its membranotropic values (Pérez-Berná et al., 2006). The authors identified the sequence (715–746) as one of the most hydrophobic regions of E2 and corresponds to the *C*-terminal transmembrane domain of E2. The region (710–725) was then synthesized and found to exhibit impressive properties including a lack of cytotoxicity, inhibition of the entry of HCVcc (HCV replicons derived from cell culture) into hepatocytes, suppression of HCV Ribonucleic acid (RNA) replication and blocking of CD81-mediated HCV entry (Liu et al., 2016).

#### Conclusions

**FIGURE 8:**
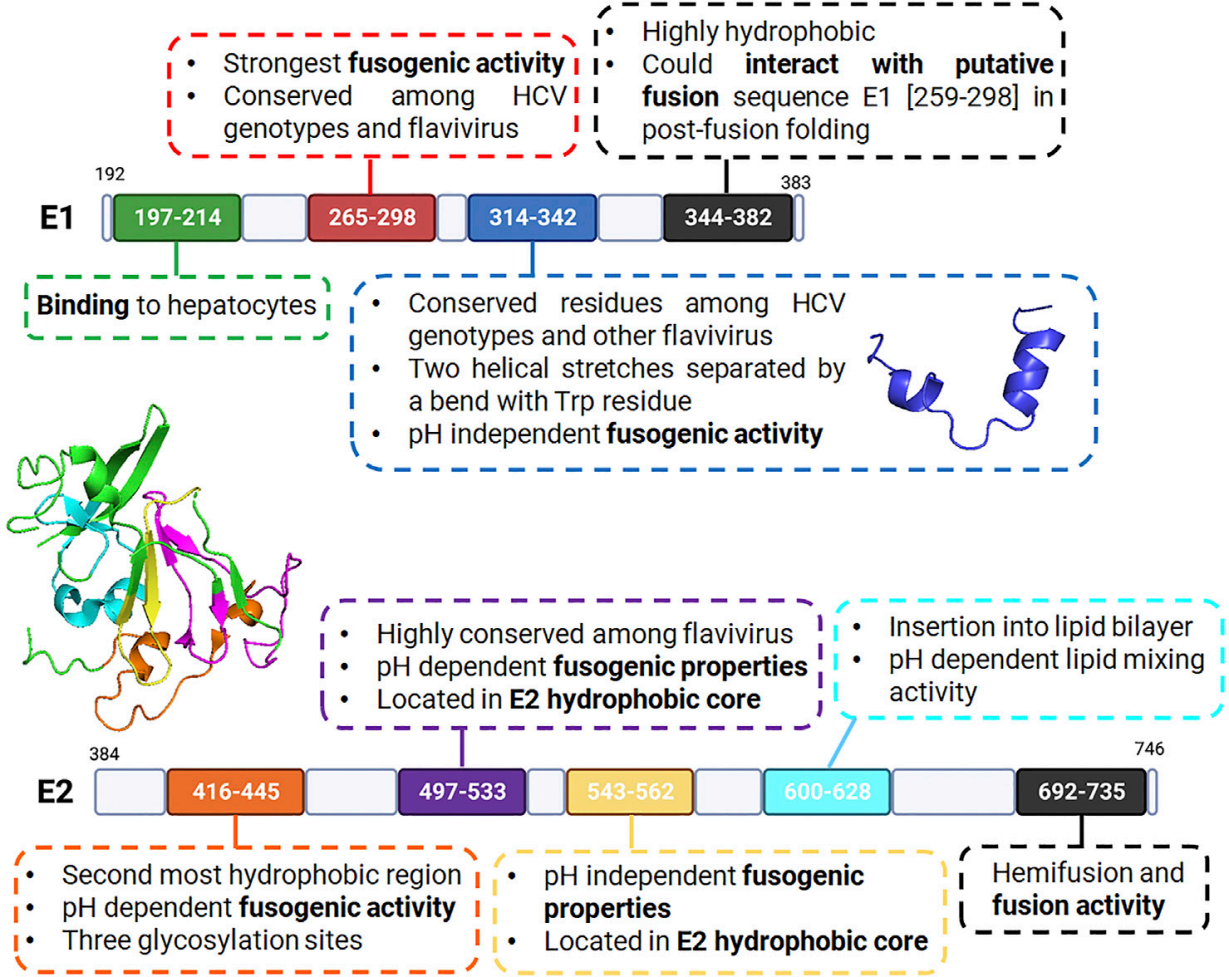
Schematic representation of HCV E1 and E2 fusion proteins with selected membrane active sequences, and their corresponding properties according to literature. Upper panel: HCV E1 protein with *N*-terminal domain (green), putative fusion peptide (red), pre-transmembrane domain (blue) and its structure determined by Spadaccini *et al.* (PDB code 2KNU) and *C*-terminal region including the transmembrane domain (black). Lower panel: HCV E2 protein and its partial ectodomain structure (determined by Kong *et al.* (Kong et al., 2013), PDB code 4MWF) with *N*-terminal domain flanked by hypervariable regions (orange), hydrophobic core region (purple and gold), pre-transmembrane domain (cyan) and *C*-terminal region transmembrane domain (black).

This case study gives an overview on how potential membranotropic sequences can be selected from fusion proteins, involved in the complex mechanism of membrane fusion during infection, in the very specific case of HCV. Even if there is a consensus about the presence of a putative fusion peptide in E1 [residues (265–298), *Putative Fusion Peptide: Residues (265–298)*], other sequences seem to be able to interact with and destabilize membranes. In fact, some of the sequences described above are referenced as having a role in fusion in AVPdb: a database of experimentally validated antiviral peptides (Qureshi et al., 2014). Moreover, interaction between some of the selected sequences, such as between the *C-*terminal region of E1 [residues (344–382), *C*-Terminal Region of E1 (344–382)] and the putative fusion peptide, highlights the possibility of a concerted action of multiple hydrophobic peptides during the fusion process. Overall, nine sequences have been identified within the HCV fusion proteins E1 and E2 from literature over the last decades (Figure 8).

Yet, despite their membranotropic activity, some sequences are unlikely to be identified as fusion peptides due to their location within the protein structures (transmembrane domain, protein core), their expected role in receptor recognition (E1 *N*-terminal domain), their glycosylation state (E2 *N*-terminal region), *etc*. Nevertheless, knowing which sequences are important and involved in the global fusion process is crucial since it allows identification of new membranotropic peptides with possible applications as cell-penetrating agents. For instance, we recently demonstrated that a modified short sequence of the E2 *N*-terminal region (419–432) was able to interact and disturb lipidic membranes and to internalize into cells *via* endocytosis (Gonzalez et al., 2019). Moreover, sequences involved in the fusion process can also be relevant for antiviral applications, serving as new biological targets or used as templates for fusion inhibitor development. Such strategy was efficiently employed by Yin *et al.* to identify two peptides (692–706) and (696–710) within the E2 transmembrane region that could inhibit HCV infection with inhibition rates of 60–80% depending on the genotype (Yin et al., 2017).

## Conclusions and Perspectives

Fusion proteins offer specificity to the membrane fusion process by providing spatial and temporal limitation of the process at cell surfaces, as well as by reducing the energetic barriers associated with the pivotal stages of membrane coalescence and subsequent pore formation. The precise identity and fusogenic properties of the peptide sequences within these proteins remain a matter of much debate. Initially, fusion peptides were difficult to define but the number of defining features increased—in line with an increased level of understanding about these peptides, which are often described as hydrophobic, conserved and flexible—and led to the identification of a larger number of peptides with fusogenic properties. To date, a well-defined definition of a fusion peptide has yet to be made. The identification of fusion peptide sequences, within the overall fusion protein, can be achieved using a number of methods that rely on specific criteria. These criteria include the hydrophobicity of a given sequence since, in order to function effectively, fusion peptides are generally considered to be the most hydrophobic segment of the whole fusion protein. In this discussion, we have also shown that fusion peptides exhibit a high degree of sequence conservation, which can be particularly pronounced among viruses of the same family.

Finally, the flexibility of these peptides is of note. Until recently, the prevailing notion was that fusion peptides adopt helical structures but, on the basis of current experimental data, it is now thought they assume a certain degree of plasticity and can resultingly adopt a mix of α- and β-like secondary structures.

Their principal role is to mediate the fusion of viral membranes with those of the host cell, as they constitute the “*active center*” of the viral fusion proteins (Nieva and Agirre, 2003). Now, though, it is suggested that several distinct sequences of these viral fusion proteins are all implicated in the process, meaning that the viral fusion process is a concerted action of different segments of the fusion proteins. Usually, a single fusion peptide is defined within a viral fusion protein, though it has been observed for several viruses that there may actually be more than one membranotropic segment within a fusion protein.

A great deal of research effort has been devoted to the definition, characterization and functional exploration of these fusion peptide sequences, in particular using X-ray crystallography and protein NMR studies, has contributed greatly to our fundamental understanding of the process (Yamauchi and Helenius, 2013; White and Whittaker, 2016). The development of new approaches to study the rapid (and energetically unfavorable) conformational changes seen in membrane-interacting proteins remain a significant challenge, but will no doubt be overcome in the near future. These, in turn, open prospective avenues for therapeutic innovation that capitalizes on these fundamental discoveries (Vigant et al., 2015; Pattnaik and Chakraborty, 2020). One can easily see the value of such discoveries in the design of new fusion inhibitors (one need only consider the efforts that have been invested in the development of an entry inhibitor for the SARS-Cov-2 virus).

Beyond preventing pathogenesis, discoveries in this field could contribute to the design of a means of entry into cells that can facilitate the delivery of molecules and genes. Even beyond that, using small, hydrophobic fusion peptides is as the vector itself by which drug molecules could be delivered. In this latter technology, small and hydrophobic fusion peptides can be compared to already known peptide drug carriers, such as the cell penetrating agents (CPP) or antimicrobial peptides (AMP) which are both cationic (Galdiero et al., 2012; Wadhwani et al., 2012).

Further studies on the fusion proteins or the fusion peptides could help to further clarify the complex fusion process at play, as well as the more nuanced structural and mechanistic issues that remain poorly understood. For instance, a resolution of structures of E1-E2 complexes of HCV in pre-fusion and post-fusion conformations would provide much-needed insight into the complex mechanism of fusion of HCV and help for the development of therapeutical applications such as a vaccine that has so far eluded researchers. Furthermore, a deeper understanding of the HCV entry process may shed light on the fusion mechanisms used by other viruses that could be, and, given what we have seen, is probably, potentially similar. Other studies for therapeutic applications have already been performed on fusion peptides showing their potential as excellent drug candidates owing to their propensity to interact with membranes either directly or indirectly (*i.e.,* by modulating a membranotropic protein) (Falanga et al., 2018).

Nevertheless, Enfuvirtide (Fuzeon™), an HIV fusion inhibitor, has surpassed US $1 billion in sales, thus showing the scale of achievement that is still ready to be harnessed by a more refined and comprehensive understanding of these hydrophobic peptide sequences, how they work and how to find them.

## Glossary

AcMNPV: autographa californica multiple nucleopolyhedrovirus
ASLV: avian sarcoma and leucosis virus
BLV: bovine leukemia virus
BVDV: bovine viral diarrhea virus
CD: circular dichroism
CD81: cluster of differentiation 81
CHIKV: chikungunya virus
CPP: cell penetrating agents
DAA: direct-acting antiviral
DLS: dynamic light scattering
DMSO: dimethyl sulfoxide
DNV: dengue virus
DPC: dodecylphosphocholine
FIV: feline immunodeficiency virus
FP: fusion peptide
FRET: förster resonance energy transfer
FTIR: fourier transform infrared
HA: hemagglutinin
HCV: hepatitis c virus
HCVcc: hepatitis c virus cell culture
HeV: hendra virus
HIV-1: human immunodeficiency virus type 1
HSV: herpes simplex virus
HTLV-1human t-cell leukemia virus type 1
HVEM: herpesvirus entry mediator
HVR: hypervariable region
iHHM: interfacial helical hydrophobic moment.
JE: japanese encephalitis
LUV: large unilamellar vesicle
MARV: marburg virus
MD: molecular dynamic
MERS-CoV: middle east respiratory syndrome-associated coronavirus
NBD: nitrobenzoxadiazole
NDV: newcastle disease virus
NMR: nuclear magnetic resonance
NTD: n-terminal domain
PFP: putative fusion peptide
RNA: ribonucleic acid
RQA: recurrence quantification analysis
SARS-CoV: severe acute respiratory syndrome-associated coronavirus
SDS: sodium dodecyl sulfate
SFV: semliki forest virus
SIV: simian immunodeficiency virus
SLE: saint louis encephalitis
SNARE: soluble *n*-ethylmaleimide-sensitive factor attachment receptor
ssRNA: single stranded RNA
SV5: simian virus 5
TBEV: tick-borne encephalitis virus
TIRF: total internal reflection fluorescent
TMD: transmembrane domain
VSV: vesicular stomatitis virus
WHO: world health organization
WNE: west nile encephalitis
WWIHS: wimley-white interfacial hydrophobicity scale
YF: yellow fever
6-HB: 6-helix bundle.
